# Intramolecular interactions and the neutral loss of ammonia from collisionally activated, protonated ω-aminoalkyl-3-hydroxyfurazans

**DOI:** 10.1177/14690667231214672

**Published:** 2023-11-17

**Authors:** J. Stuart Grossert, Donatella Boschi, Marco L. Lolli, Robert L. White

**Affiliations:** 1 Department of Chemistry, 98609 Dalhousie University, Halifax, Nova Scotia, Canada; 2 Dipartimento di Scienza e Tecnologia del Farmaco (DSTF), 9314 Università degli Studi di Torino, Torino, Italy

**Keywords:** Collisional activation, DFT computations, ESI(+)-MS/MS, fragmentation mechanisms, heterocycles, 3-hydroxyfurazan

## Abstract

Gas phase fragmentation reactions of monoprotonated 4-(3-aminopropyl)- and 4-(4-aminobutyl)-3-hydroxyfurazan were investigated to examine potential interactions between functional groups. The two heterocyclic alkyl amines were ionized by electrospray ionization (ESI, positive mode) and fragmented using tandem mass spectrometry (MS/MS). The fragmentation pathways were characterized using pseudo MS^3^ experiments, precursor-ion scans, and density functional computations. For both heterocyclic ions, loss of ammonia was the only fragmentation process observed at low collision energies. Computational analysis indicated that the most feasible mechanism was intramolecular nucleophilic displacement of ammonia from the protonated ω-aminoalkyl side chain by N5 of the furazan ring. The alkylated nitrogen in the resulting bicyclic product ion facilitated N-O bond cleavage; subsequent neutral losses of nitric oxide (NO) and carbon monoxide (CO) occurred by homolytic bond cleavages. Next in the multistep sequence, neutral loss of ethylene from a radical cation was observed. A less favorable, competing fragmentation pathway of protonated 4-(3-aminopropyl)-3-hydroxyfurazan was consistent with cleavage of the 3-hydroxyfurazan ring and losses of NO and CO. Overall, the similar fragmentation behavior found for protonated 4-(3-aminopropyl)- and 4-(4-aminobutyl)-3-hydroxyfurazan differed from that previously characterized for furazan analogs with shorter alkyl chains. These observations demonstrate that a small change in the structure of multifunctional, heterocyclic alkyl amines may significantly influence interactions between distinct functional groups and the nature of the fragmentation process.

## Introduction

The rings in the structures of biologically active heterocycles, such as pharmaceuticals,^1–3^ antifungal agents,^4,5^ and herbicides,^6^ provide both a rigid scaffold and hydrogen bonding sites.^7–10^ When analyzed by tandem mass spectrometry (MS/MS), heterocyclic ions often fragment by a prominent and characteristic ring cleavage^11–18^ or by cleavage of a bond to the ring.^19^ In other instances, the ring participates in proton transfer prior to the dissociative step and is located intact in the neutral product or the product ion.^20–24^ In general, distinctive gas phase, MS/MS fragmentation processes are valuable for the identification and structure elucidation of trace amounts of substances as well as for quantitative determinations using selected or multiple reaction monitoring (SRM/MRM) techniques.^25,26^

Heterocycles containing a hydroxy substituent and three contiguous heteroatoms in a five-membered ring have been recognized as bioisosteres of the carboxyl group and then utilized as a drug design strategy.^7,8,27–36^ Accordingly, 3-hydroxyfurazan heterocycles have been incorporated into the structures of glutamate analogs,^28,32^ dihydroorotate dehydrogenase inhibitors,^30^ as well as anticancer^33,35^ and immunosuppressive agents.^34^

The aminomethyl- and 2-aminoethyl 3-hydroxyfurazans **1** and **2** were readily protonated by electrospray ionization mass spectrometry (ESI MS, positive mode) and fragmented by ring cleavage upon collision-induced dissociation (CID).^37^ In the lowest energy pathway, ring cleavage was accompanied by neutral losses of nitric oxide (NO) and carbon monoxide (CO). At higher collision energies, other fragmentation pathways, including an initial loss of ammonia, were observed as well. The three nitrogen atoms in 4-(ω-aminoalkyl)-3-hydroxyfurazans **1a**(ol) and **2a**(ol) are potential protonation sites, and the heterocycles may also exist as the higher energy tautomeric, lactam forms **1a**(one) and **2a**(one). Computational analyses were used to associate the initial step of each pathway with a particular tautomer protonated at a specific site. For example, protonation at N5 led to ring cleavage and successive losses of NO and CO.

**Figure 1.**
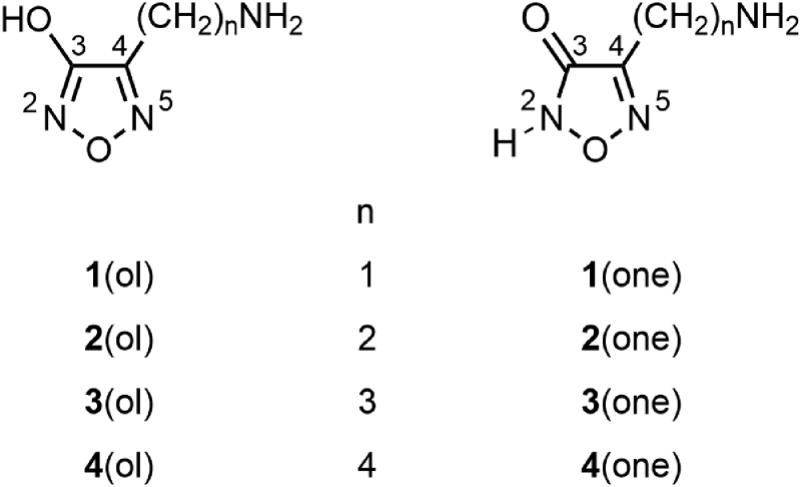
Tautomeric 4-(ω-aminoalkyl)-substituted 3-hydroxyfurazans (1,2,5-oxadiazol-3-ols) and 1,2,5-oxadiazol-3(*2H*)-ones. The corresponding [M + H]^+^ ions are designated as **1a**–**4a** (mixture of tautomers) or specifically as lactim tautomers, **1a**(ol)–**4a**(ol), and lactam tautomers, **1a**(one)–**4a**(one).

In the current investigation, the fragmentation pathways of protonated 4-(3-aminopropyl)- and 4-(4-aminobutyl)-3-hydroxyfurazans (**3a** and **4a**, respectively, Figure 1) were studied using tandem mass spectrometry and density functional theory (DFT) computations. Both cations contain two different functional groups linked by an alkyl chain. Either the primary amine or a tautomer of the heterocycle may be protonated in the gas phase^37^ and fragmentation may or may not occur through intramolecular interactions. Unlike the fragmentation by furazan ring cleavage documented for protonated 4-(aminomethyl)- and 4-(2-aminoethyl)-3-hydroxyfurazan (**1a** and **2a**, respectively, Figure 1),^37^ the prominent fragmentation process found for the homologous ions **3a** and **4a** was the loss of ammonia. This favorable process was attributed to intramolecular displacement of the protonated primary amino group by a nitrogen atom in the ring. Ring cleavage occurred as a secondary process for both protonated furazans and as a competing fragmentation process of **3a** at higher collision energies.

## Materials and methods

The 4-(ω-aminoalkyl)-3-hydroxyfurazans (**3** and **4**) were available from previous studies.^27^ The hydrochloride salts of **3** and **4** were dissolved in aqueous methanol (1 mg mL^–1^) and a portion (5–10 μL) was introduced into the spectrometer by flow injection (H_2_O:MeOH: 1:1, v/v, 20 μL min^–1^).

Gas phase ions were generated by positive-ion electrospray (ESI(+)) and mass spectra were acquired on Thermo-Finnigan LCQ-DUO ion trap and Micromass Quattro triple quadrupole mass spectrometers. Instrument settings^38,39^ are provided in the supplemental material (Tables S1 and S2). Pseudo MS^3^ spectra and precursor-ion scans were acquired after an ion (e.g. **3a**) was subjected to non-selective, in-source fragmentation. In-source ions were then mass selected for CID in the ion trap or the collision cell of the triple quadrupole mass spectrometer (MS/MS) to generate pseudo MS^3^ spectra. In the triple quadrupole mass spectrometer, precursor-ion scans were collected by setting the third quadrupole to select a certain product ion and by scanning ions from the source in the first quadrupole to locate the precursors that dissociate to the selected product ion. Accurate masses were measured on a Bruker Daltonic Compact QToF spectrometer.^38^

**Figure 4.**
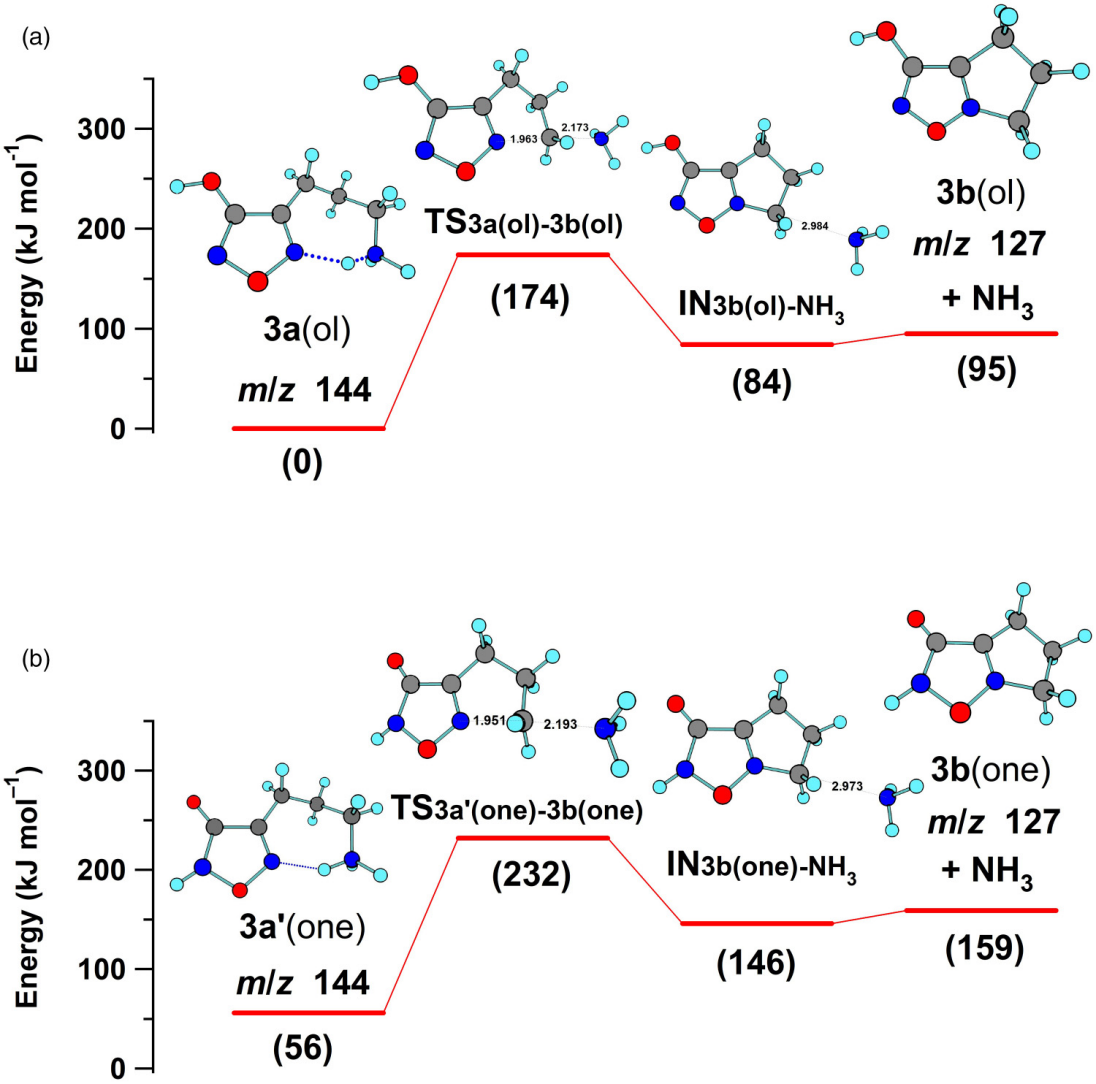
Potential energy profiles computed for the nucleophilic displacement of ammonia from the protonated tautomers of 4-(3-aminopropyl)-3-hydroxyfurazan **3a**(ol) (a) and **3a**(one) (b).

The energetics of fragmentation processes were computed using DFT and Møller-Plesset perturbation theory (MP2) within the Gaussian 09 suite of programs (Revision C.01).^40^ Geometry optimizations and frequency calculations were performed using the ωB97X-D/6-311 + G(d) functional.^41^ Energy minima were characterized by having no imaginary vibrational frequencies, whereas saddle points had one such frequency. Thermochemical data are reported as combinations of single-point MP2/6-311++G(2d,p) electronic energies and uncorrected entropies and thermal corrections from the ωB97X-D/6-311 + G(d) calculations and are designated as MP2/6-311++G(2d,p)//ωB97X-D/6-311 + G(d) free energies given in kJ mol^–1^. Higher energy conformations of a particular ion located computationally are denoted by the addition of a prime to the ion descriptor (e.g. **3aʹ**(one) in Figure 4(b)). Cartesian coordinates for the optimized structures are given in the supplemental material.

## Results and discussion

**Figure 2.**
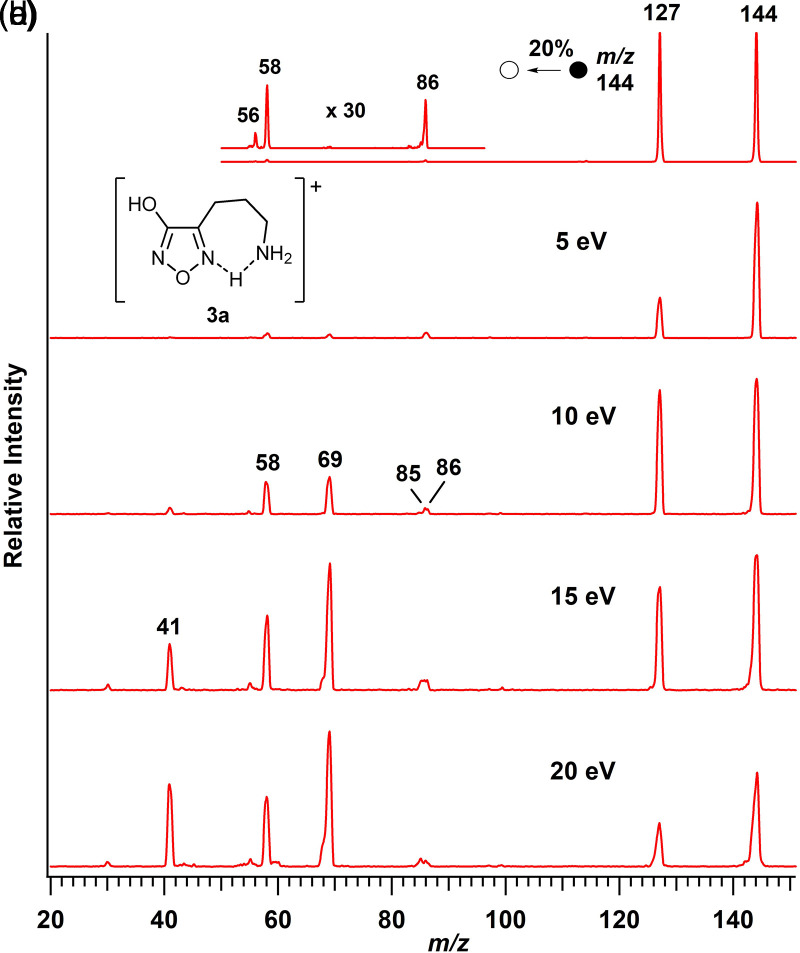
Tandem mass spectra of protonated 4-(3-aminopropyl)-3-hydroxyfurazan (**3a**, *m*/*z* 144, [M + H]^+^) collected on an ion trap mass spectrometer (a) and on a triple quadrupole mass spectrometer (15 V cone) over a range of collision energies (b–e).

The ω-aminoalkyl heterocycles 4-(3-aminopropyl)-3-hydroxyfurazan (**3**) and 4-(4-aminobutyl)-3-hydroxyfurazan (**4**) were protonated readily when subjected to ESI. In the ion trap mass spectrometer and at low collision energy (5–10 eV, lab. frame) in the triple quadrupole mass spectrometer, the tandem mass spectra of **3a** (*m*/*z* 144, Figure 2) and **4a** (*m*/*z* 158, Figure S1) showed prominent product ions at *m*/*z* 127 and *m*/*z* 141, respectively, corresponding to a neutral loss of NH_3_. Accurate mass determinations by MS/MS (*m*/*z* 144.0776 → *m*/*z* 127.0507 and *m*/*z* 158.0921 → *m*/*z* 141.0656) confirmed the loss of NH_3_. At higher collision energies (15–20 eV) in the triple quadrupole mass spectrometer (Figure 2, spectra (d) and (e); Figure S1, spectra D and E), the formation of lower mass product ions (< *m*/*z* 110) indicated more extensive fragmentations of **3a** and **4a**.

### Protonation site and initial neutral loss of ammonia

The relative energies of ions **3a** and **4a** differing in protonation site and conformation were determined computationally (Table S3). Ions protonated at N5 have higher energy and the lowest energy ions **3a**(ol) and **4a**(ol) were protonated on the primary amino group, the most basic site. The ions were stabilized by intramolecular hydrogen bonds and, typically, conformations of the lactim tautomer were more stable than the corresponding lactam tautomer. Overall, the relative energies of the ions were equivalent to those computed for the shorter chain protonated ω-aminoalkyl-3-hydroxyfurazans (**1a** and **2a**).^37^ Note that protonation of the primary amino group created structural connections matching the structure of ammonia.^37^

**Figure 3.**
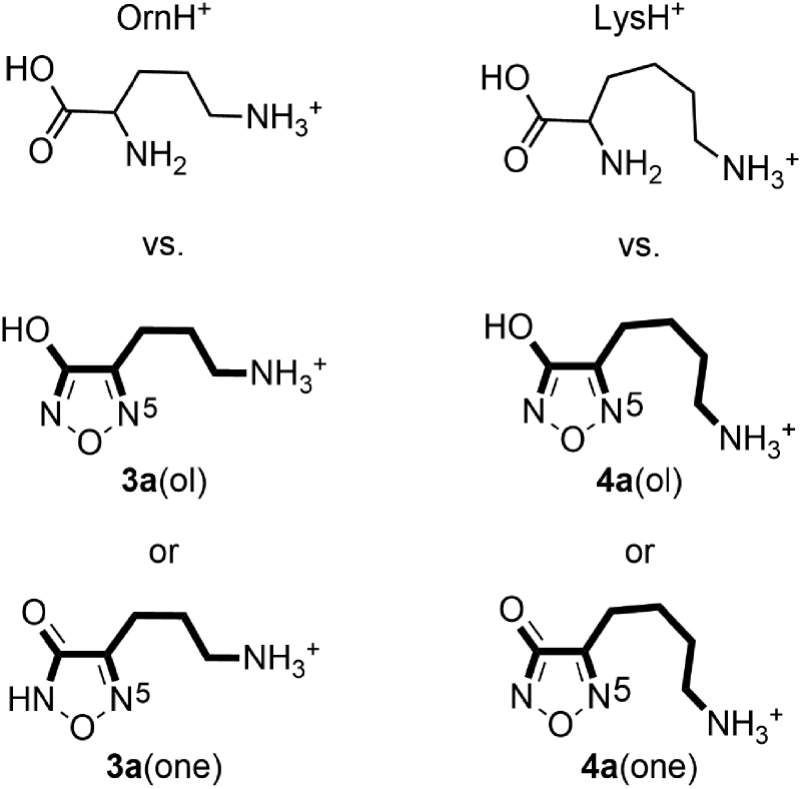
Structural comparison of protonated ornithine (OrnH^+^) with protonated 4-(3-aminopropyl)-3-hydroxyfurazan, **3a**(ol), and its tautomer 4-(3-aminopropyl)-1,2,5-oxodiazol-3(*2H*)-one, **3a**(one). The portion of the aminoalkyl heterocycle corresponding to the structure of the diamino acid is highlighted. The analogous structural relationship is found for the protonated homologs lysine (LysH^+^), 4-(4-aminobutyl)-3-hydroxyfurazan, **4a**(ol), and 4-(4-aminobutyl)-1,2,5-oxodiazol-3(*2H*)-one, **4a**(one).

The loss of ammonia from the terminal position of an aliphatic chain has been documented for several protonated, structural analogs, such as diaminoalkanes,^42^ diamino, dicarboxylic acids,^38,42^ aminoalcohols,^43^ muscimol, an aminomethyl substituted hydroxyisoxazole,^44–46^ and amlodipine, an aminoethoxymethyl substituted dihydropyridine calcium channel blocker.^47,48^ In particular, the structures of both tautomers of the 3-hydroxyfurazans **3a** and **4a** closely resemble the structures of the monoprotonated forms of the common, naturally occurring diamino acids ornithine (OrnH^+^) and lysine (LysH^+^) (Figure 3). Indeed, the 3-hydroxyfurazan ring is a conformationally constrained analog of the α-amino and carboxyl groups, whereas the ω-aminoalkyl substituents in **3a** and **4a** are identical to the side chains of ornithine and lysine, respectively. Upon MS/MS, the prominent loss of the side chain nitrogen from OrnH^+^ and LysH^+^ was confirmed by isotopic labeling, and computations showed that the nucleophilic displacement of the protonated, side chain amino group by the α-amino group of OrnH^+^ to form a favorable five-membered ring was feasible.^38^

In the current investigation, a reasonable energy (174 kJ mol^–1^) was computed for the cyclic five-membered transition structure **TS3a**(ol)**-3b**(ol) formed upon loss of ammonia from the most stable conformation of **3a**(ol) (Figure 4(a)). This displacement of ammonia from the aminopropyl side chain by N5 in the ring of **3a** is analogous to the process characterized for OrnH^+^ (computed barrier = 153 kJ mol^–1^).^38^ The potential energy profile was also computed for **3aʹ**(one), the higher energy and probably less abundant tautomer in the gas phase. Although the profile was shifted to higher energy (Figure 4(b)), a similar barrier (176 kJ mol^–1^) was computed for the corresponding N5 nucleophilic displacement of ammonia. Overall, the tautomers **3a**(ol) and **3aʹ**(one) gave tautomeric, bicyclic product ions (**3b**(ol) and **3b**(one)).

A similar intramolecular, nucleophilic displacement of NH_3_ from the 4-aminobutyl furazan **4a** would form a favorable six-membered ring. The corresponding six-membered ring formation upon the loss of ammonia has been characterized by mass spectrometry for the fragmentation of LysH^+^,^38^ which also has a 4-aminobutyl group (Figure 3). With the loss of ammonia as the predominant, initial fragmentation process observed for **4a** and LysH^+^ and the structural correlation of the ions, the evidence is consistent with fragmentation of both ions by the same displacement mechanism.

Although the computations indicate reasonable energetics for the nucleophilic displacement of ammonia, other possible mechanisms were investigated, such as elimination processes. A barrier of 216 kJ mol^–1^ was computed for the elimination of ammonia from the 3-hydroxyfurazan **3aʹʹ**(ol) via abstraction a proton from the propyl side chain by N5 (Figure S2A). For the analogous proton abstraction and elimination of ammonia from the tautomer **3aʹʹʹʹ**(one), the potential energy profile (Figure S2B) was shifted to higher energy, but the height of the barrier was similar (230 kJ mol^–1^). On the other hand, proton abstraction by the exocyclic, carbonyl oxygen required only 157 kJ mol^−1^ to effect the loss of ammonia from a higher energy conformation (**3aʹʹʹ**(one)) of the lactam tautomer (Figure S3). The structures of the *m*/*z* 127 product ions resulting from these elimination processes differ in the placement of protons on two of three heteroatoms (i.e. N2, N5 and the exocyclic oxygen).

Each of the four mechanisms considered above for the loss of ammonia from **3a** required similar inputs of energy but generated a product ion with a distinct structure, namely **3b**(ol), **3b**(one), **3c**(ol), **3c**(one), and **3c**_p_. Thus, the secondary fragmentation reactions of **3a** were examined to distinguish the ions formed by the initial loss of ammonia and its mechanism.

### Fragmentation pathways of 3a

When **3a** was subjected to CID at higher collision energies (10–20 eV, lab. frame, MS^2^) in the triple quadrupole mass spectrometer (Figures 2(c)–2(e)), the primary product ion at *m*/*z* 127 was accompanied by major product ions at *m*/*z* 69, 58, and 41 and minor product ions at *m*/*z* 86 and 85. Precursor-product ion relationships were established for these more extensive fragmentations by pseudo MS^3^ spectra (Figure S4A), and by precursor-ion scans (Figure S4B). Thus, the fragmentation sequence *m*/*z* 144 → 127 → 97 → 69 → 41 was established as the most important pathway of **3a**, while the sequence *m*/*z* 144 → 86 → 58 → 41 was identified as a competing pathway.

**Scheme 1.**
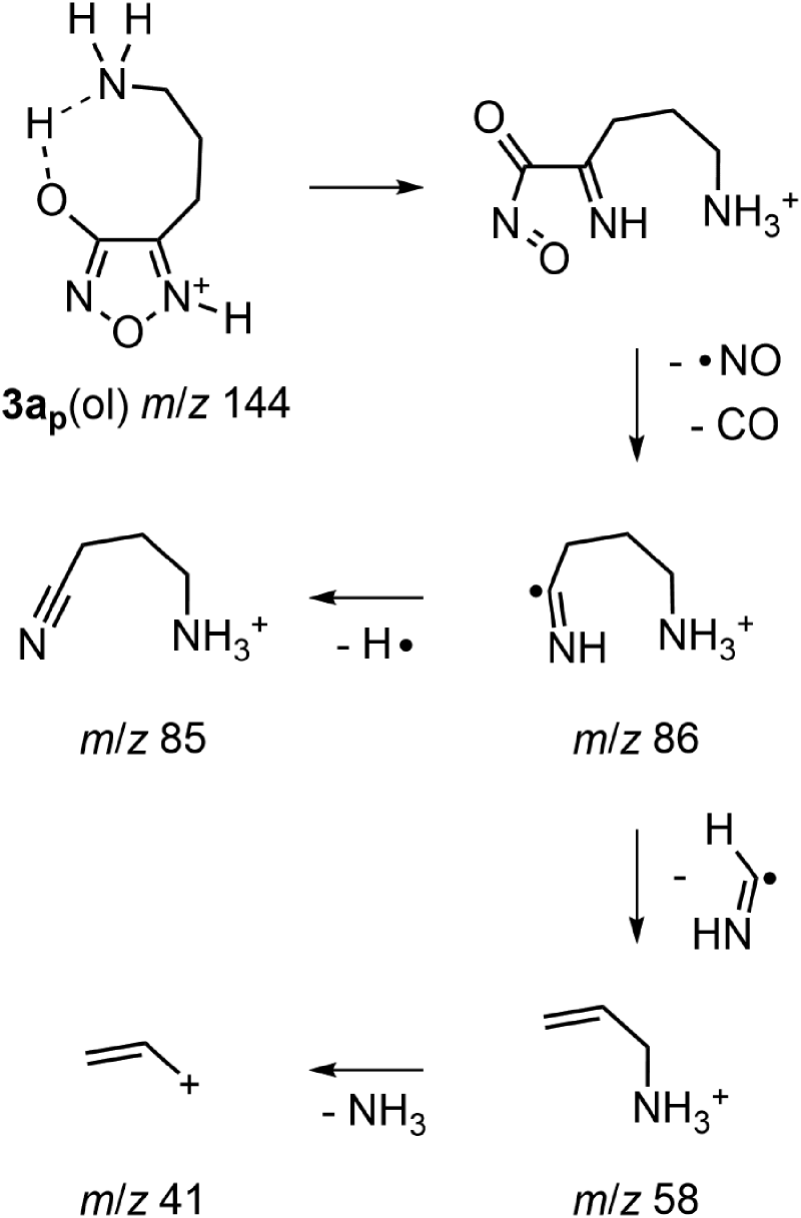
Competing fragmentation pathway of protonated 4-(3-aminopropyl)-3-hydroxyfurazan (**3a_p_**(ol), *m*/*z* 144) by initial ring cleavage.

For the competing pathway, the initial neutral loss of 58 u (*m*/*z* 144 → 86; Figures 2 and S4B) corresponded to the combined neutral loss of NO and CO. The analogous transition has been characterized as the lowest energy fragmentation process for protonated 4-(aminomethyl)-3-hydroxyfurazan (**1a**).^37^ The bond connectivity in the 3-hydroxyfurazan requires ring cleavage to explain the formation of NO and CO. Previous computations on **1a** indicated that ring opening by N5–O bond cleavage was achieved with input of modest energy when N5 was protonated and the proton on oxygen was abstracted by the side chain amino group.^37^ Accordingly, protonation at N5 and abstraction of the oxygen proton by the primary amino group gave the analogous ring opening of **3a_p_**(ol) (Scheme 1). Subsequent homolytic bond cleavage generated NO (a neutral radical), CO and the radical cation (HN = C^•^–CH_2_CH_2_CH_2_NH_3_^+^) observed at *m*/*z* 86.

In the precursor-ion scans (Figure S4B), the ion at *m*/*z* 58 was connected to ions at *m*/*z* 86 and 144 (**3a**), and the ion at *m*/*z* 41 was linked to the ion at *m*/*z* 58 and to other higher mass ions. Thus, the *m*/*z* 86 ion is a likely source of both the ion at *m*/*z* 58 (H_2_C = CHCH_2_NH_3_^+^) and the minor ion at *m*/*z* 85 (N≡CCH_2_CH_2_CH_2_NH_3_^+^) (Figures 2(c)–2(e) and S4B, middle spectrum) by respective losses of a methanimine radical (HN = CH•) and a hydrogen atom (Scheme 1). The possible conversion of *m*/*z* 58 to *m*/*z* 41 is consistent with loss of ammonia by simple C–N bond cleavage.

In the main fragmentation pathway of **3a**, the initial neutral loss of ammonia (*m*/*z* 144 → 127, Figures 2(a) and 2(b)) was followed by successive neutral losses of NO and CO (58 u) from **3b** (*m*/*z* 127 → 97 → 69, Figure S4). The latter was also observed in the first step in the competing pathway of **3a** (*m*/*z* 144 → 86, Scheme 1) and as the lowest energy fragmentation pathway of protonated 4-aminomethyl-3-hydroxyfurazan (**1a**).^37^ In these pathways, the development of a formal positive charge on N5 by protonation was the key determinant for ring cleavage. In the nucleophilic displacement of ammonia from **3a** (Figure 4), alkylation generated a formal positive charge on N5, enabling N5–O bond cleavage and the subsequent loss of NO and CO.

**Figure 5.**
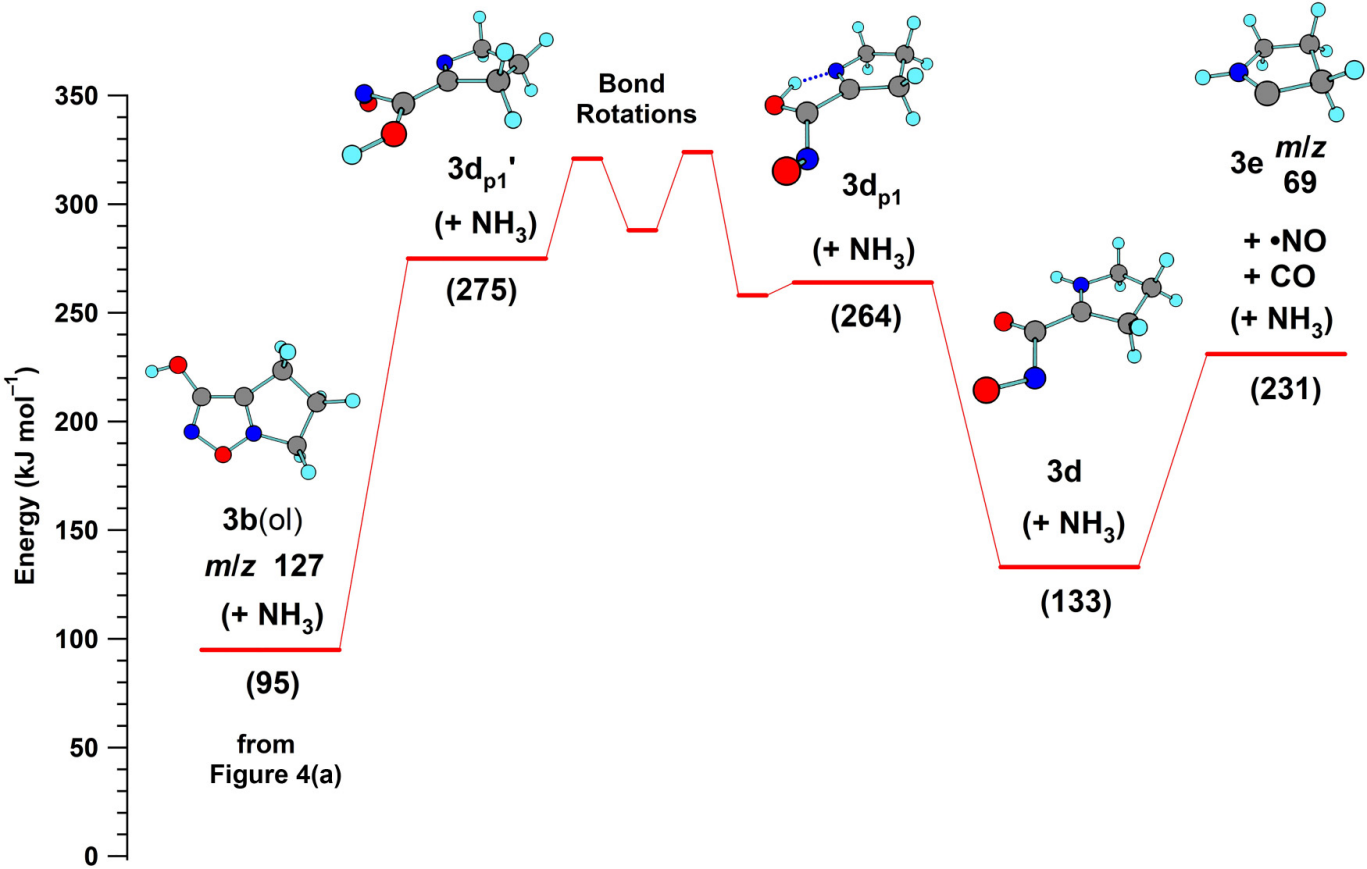
Potential energy profile for the fragmentation of **3b**(ol) (*m*/*z* 127) showing ring opening and subsequent loss of NO and CO yielding the radical cation **3e** (*m*/*z* 69).

The energy computed for the endergonic ring cleavage (**3b**(ol) → **3d_p1_ʹ**, 180 kJ mol^–1^, Figure 5) was similar to that found for the nucleophilic displacement of ammonia (Figure 4). Subsequent low-energy conformational changes gave a suitable alignment for the exergonic abstraction of the proton on oxygen by nitrogen and the formation of a more stable iminium ion (**3d**). Homolytic bond cleavage with the release of NO and CO and formation of a cyclic radical cation (**3e**, *m*/*z* 69) had a modest barrier of 98 kJ mol^–1^. Cleavage of the ring in **3e**, which was formed by the initial displacement of ammonia, led to the formation of a radical cation at *m*/*z* 41 (**3f**, HN ^+ ^≡C–CH_2_•, Figure S4A) and the loss of ethylene. The sequence of reactions for the fragmentation of **3a**(ol) to **3f** is shown in Scheme S1.

Thus, an energetically feasible pathway for the fragmentation of **3a**(ol) to **3e** (*m*/*z* 69) was supported by the computations (Figures 4(a) and 5). However, a parallel pathway consisting of analogous fragmentation reactions of the higher energy lactam tautomer **3aʹ**(one) is also mechanistically reasonable (Scheme S1). The energetic feasibility of the initial loss of ammonia from **3a**(one) was shown by computations (Figure 4(b)) and, as outlined in Scheme S1, ring cleavage of **3b**(one) followed by proton transfer generates **3d**, the ion also formed by rearrangement of **3b**(ol). Consequently, **3aʹ**(one) is a possible, but higher energy, starting point for a second fragmentation pathway for the formation of product ion **3d** (*m*/*z* 127) and the subsequent formation of the lower mass product ions **3e** (*m*/*z* 69) and **3f** (*m*/*z* 41).

Another possibility for the loss of ammonia is by an elimination mechanism. The abstraction of a proton from the propyl side chain by N5 in **3aʹʹ**(ol) generates ion **3c**(ol), a protonated allyl-substituted furazan (Figure S2A). Subsequent fragmentation of **3c**(ol) by ring cleavage and losses of NO and CO is mechanistically possible (Scheme S2). As seen in the pathways initiated by nucleophilic displacement of ammonia (Figures 4 and 5; Scheme S1), a proton transfer step is needed before the loss of NO and CO occurs to form an acyclic radical cation (CH_2 _= CH–CH_2_–C^•^=NH_2_^+^) at *m*/*z* 69. A parallel sequence of analogous reactions for the fragmentation of **3aʹʹʹʹ**(one) to the *m*/*z* 69 ion is also mechanistically feasible (Scheme S2). Homolytic C–C bond cleavage of the radical cation (*m*/*z* 69) produces an ion-neutral complex from which loss of ethylene requires hydrogen atom abstraction. If fragmentation of **3a** proceeded by the routes outlined in Scheme S2, the formation of an ion at *m*/*z* 42 by dissociation of the ion-neutral complex and an ion at *m*/*z* 68 by loss of a hydrogen atom (see next section) is possible. However, neither ion was detected in the mass spectra of **3a** (Figures 2 and S4A). The latter, along with the higher energy input computed for the initial elimination step (Figure S2A), suggest that this reaction sequence makes a smaller or negligible contribution to the fragmentation of **3a**.

The elimination of ammonia by proton abstraction by the carbonyl oxygen in **3a**(one) was studied computationally (Figure S3). Although a barrier of only 157 kJ mol^–1^ was located, the computations indicated that the elimination proceeded from **3aʹʹʹ**(one). This higher energy conformation of the lactam tautomer is most likely a very minor component of the cations generated by ESI. Also, the energy required for the separation of ammonia from the product ion **3c_p_** (*m*/*z* 127) was somewhat large (99 kJ mol^–1^). The proton in **IN3c-NH_3_** is much closer to N than O and dissociation to the complementary^3^ ion NH_4_^+^ (*m*/*z* 18) and a neutral heterocycle may be favored. However, the ion **3c_p_** produced by this elimination of ammonia is protonated at N2 of the ring. Unlike its protomer **3c**(ol), neutral losses of NO and CO upon ring cleavage of **3c_p_** is less likely and the formation of **3c_p_** is therefore a poor fit with the experimental observations.

In principle, nucleophilic displacement of ammonia by the exocyclic oxygen is possible. In this instance, both the additional bond to oxygen and the location of the formal positive charge are incompatible with ring cleavage leading to the loss of both oxygen atoms in **3a**(ol) and **3a**(one) as NO and CO. As a result, the products formed by these mechanisms are a poor match with the experimental observations (Figures 2 and S4) and the reactions involving the exocyclic oxygen atom are unlikely to contribute to the loss of ammonia.

Overall, the analysis of secondary fragmentation processes and computed energetics indicate that the observed initial loss of ammonia occurs from **3a**(ol) upon nucleophilic displacement by N5 in the ring.

### Fragmentation pathway of 4a

In accord with the structural relationship between protonated 4-(4-aminobutyl)-3-hydroxyfurazan (**4a**) and **3a** (Figure 1), the tandem mass spectra collected for **4a** (*m*/*z* 158; Figure S1) showed product ions at *m*/*z* 141, 83, and 55 that are homologs of ions observed in the mass spectra of **3a** (Figures 2 and S4). The sequence of neutral losses (NH_3_, NO, CO and ethylene) observed for **4a** was the same as that found in the main fragmentation pathway of **3a** (Scheme S1), and the extension of the alkyl chain by one methylene unit had little or no effect on the fragmentation behavior of the homologous ion.

**Scheme 2.**
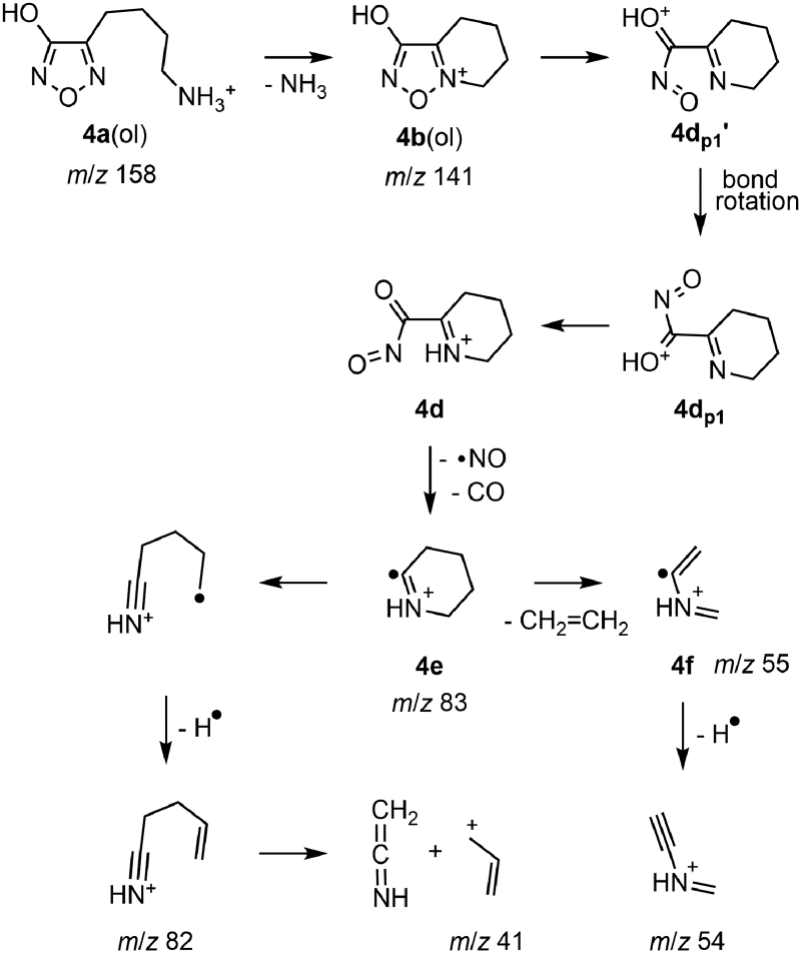
A pathway for the fragmentation of protonated 4-(4-aminobutyl)-3-hydroxyfurazan (**4a**(ol)) showing the formation of the radical cation **4e** by sequential neutral losses of NH_3_, NO and CO. Subsequent fragmentation of **4e** occurred either by loss of CH_2_ = CH_2_ and a hydrogen atom or by the loss of a hydrogen atom and CH_2_ = C = NH.

The similar relative energies computed for the various **4a** and **3a** ions (Table S3) suggested that the analogous **4a** ions were likely starting points for the fragmentation pathways characterized for **3a** (vide supra). Thus, fragmentation of **4a**(ol) by the reaction sequence described for **3a** (Figures 4(a) and 5; Scheme S1) led to a cyclic radical cation (**4e**) at *m*/*z* 83 (Scheme 2). Subsequent loss of ethylene gave the product ion at *m*/*z* 55 (**4f**). In the pseudo MS^3^ spectrum of *m*/*z* 141 (Figure S1, spectrum A inset) and at high collision energies (15–20 eV; Figure S1, spectra D and E), ions at *m*/*z* 82 and 54 accompanied the ions at *m*/*z* 83 and 55 indicating losses of a hydrogen atom. This loss of a neutral atom is consistent with the radical cation structures proposed for the ions at *m*/*z* 83 (**4e**) and 55 (**4f**).

As an alternative to the loss of ethylene from the cyclic radical cation **3e** (Scheme S1), the neutral loss from the six-membered ring in **4e** was attributed to ring cleavage by an electrocyclic process (Scheme 2). The analogous reaction has been proposed for the loss of ethylene from the 1-piperideine ion, a cyclic fragmentation product of LysH ^+ ^.^38^

## Conclusions

The predominant, initial fragmentation process observed for both the protonated 4-(3-aminopropyl)- and 4-(4-aminobutyl)-3-hydroxyfurazans **3a** and **4a**, respectively, was the neutral loss of ammonia. Computational analysis indicated that the process occurred by intramolecular, nucleophilic displacement of the protonated primary amine by N5 of the furazan ring. In previous studies,^37^ the ring protonated 4-(aminomethyl)- and 4-(2-aminoethyl)-3-hydroxyfurazans **1a** and **2a**, respectively, fragmented by ring cleavage with successive losses of NO and CO. Although the NO/CO neutral loss was also observed in the tandem mass spectra of **3a** (Figure 2), it was observed only at higher collision energies. Thus, the homologous series of protonated heterocycles (**1a**–**4a,** Figure 1) fragmented by structure-specific processes depending on the protonation site and the length of the aminoalkyl side chain. By contrast, the anions formed by deprotonation of compounds 1–4 underwent ring cleavage upon CID with the formation of a common product ion (OCNO^–^).^49^

The computations indicated that fragmentation of the lactim tautomer **3a**(ol) was the most energetically favorable process. Although similar barriers were found for the analogous reactions of lactam tautomer **3a**(one), smaller amounts of this higher energy tautomer were likely formed by ESI.

The bicyclic product ion **3b**(ol) formed by the loss of ammonia from **3a**(ol) (Figure 4(a)) fragmented by 3-hydroxyfurazan ring cleavage with loss of NO and CO (Figure 5). In this cyclization process, a formal positive charge develops on N5 of the furazan ring and its fragmentation is analogous to the initial process characterized previously for **1a** and **2a** when protonated on N5.^37^

The present and previous^37^ computations indicated that the energy of the ion formed by protonation of the heterocyclic alkyl amine varied with protonation site, conformation, the extent of hydrogen bonding, and the tautomeric form of the heterocycle. The similar energetics provided several possibilities as starting points for fragmentation pathways and indicated that structural isomers of isobaric ions are generated during electrospray ionization. Nevertheless, the initial fragmentation behavior of the homologous heterocycles **1a**–**4a** is significantly influenced by small structural changes such as the length of the alkyl chain and the site of protonation. The results further establish the importance of considering multiple variables for the interpretation/prediction of the fragmentation pathway of ions formed from multifunctional molecules.

## Supplemental Material

sj-docx-1-ems-10.1177_14690667231214672 - Supplemental material for Intramolecular interactions and the neutral loss of ammonia from collisionally activated, protonated ω-aminoalkyl-3-hydroxyfurazans Supplemental material, sj-docx-1-ems-10.1177_14690667231214672 for Intramolecular interactions and the neutral loss of ammonia from collisionally activated, protonated ω-aminoalkyl-3-hydroxyfurazans by J. Stuart Grossert, Donatella Boschi, Marco L. Lolli and Robert L. White in European Journal of Mass Spectrometry
