# Supplementary material for: Intramolecular interactions and the neutral loss of ammonia from collisionally activated, protonated ω-aminoalkyl-3-hydroxyfurazans
Source: Eur J Mass Spectrom (Chichester). 2023 Nov 17;30(1):38–46. doi: 10.1177/14690667231214672 (PMC10809737; doi:10.1177/14690667231214672)
Supplement: sj-docx-1-ems-10.1177_14690667231214672 - Supplemental material for Intramolecular interactions and the neutral loss of ammonia from collisionally activated, protonated ω-aminoalkyl-3-hydroxyfurazans [file sj-docx-1-ems-10.1177_14690667231214672.docx]

**Intramolecular interactions and the neutral loss of ammonia from collisionally activated, protonated ω-aminoalkyl-3-hydroxyfurazans**

J. Stuart Grossert,^1^ Donatella Boschi,^2^ Marco L. Lolli^2^ and Robert L. White^1*^

1) Department of Chemistry, Dalhousie University, 6274 Coburg Road, PO Box 15000, Halifax, Nova Scotia, B3H 4R2, Canada

2) Dipartimento di Scienza e Tecnologia del Farmaco (DSTF), Università degli Studi di Torino, via Pietro Giuria 9, 10125 Torino, Italy

***Correspondence:** R.L. White; Email: robert.white@dal.ca

***Supplementary Material***

**Experimental**

Table S1 LCQ Duo mass spectrometer S2

Table S2 Micromass Quattro triple quadrupole mass spectrometer S3

**Results**

Figure S1 MS/MS spectra of protonated 4-(4-aminobutyl)-

3-hydroxyfurazan (**4a**) S4

Table S3 Structures computed for **3a** and **4a** S5

Figure S2 PE profile for the elimination of NH_3_ from **3a** S6

Figure S3 PE profile for the elimination of NH_3_ from **3aʹʹʹ**(one) S7

Figure S4 Pseudo MS^3^ mass spectra and precursor ion scans S8

Scheme S1 Fragmentation pathways of **3a**(ol) and **3a**(one) S9

Scheme S2 Fragmentation pathways of **3aʹ**(ol) and **3aʹ**(one) S10

Cartesian coordinates of atoms in computed structures S11

**Experimental**

**Table S1.** Thermo-Finnigan LCQ Duo ion trap mass spectrometer: flow injection conditions and instrument settings. Data acquisition and processing were conducted using Thermo Scientific Xcalibur software.

| solvent | H_2_O:MeOH (1:1, v/v) |
| --- | --- |
| sample concentration | 1 mg mL^–1^ |
| flow rate | 20 μL min^–1^ |
| ESI potential | 3.5 kV |
| sheath gas (N_2_) flow rate | 20 arbitrary units |
| capillary temperature | 200°C |
| maximum injection time | 200 ms |
| damping and collision gas | helium |
| source potential | 0–25 V |
| minimum *m*/*z* detected  (i.e., low mass cut-off) | 50 |
| CID isolation width | 1 u |
| collision energy | 15–30% |
| The collision energy (reported as an arbitrary percentage) was adjusted to the minimum needed to assign the base peak in the spectrum to a product ion. Typically, 10-30 scans were combined to produce spectra. | |

**Table S2.** Micromass Quattro triple quadrupole mass spectrometer: flow injection conditions and instrument settings. Data acquisition and processing were conducted using MassLynx MS software.

| solvent | H_2_O:MeOH (1:1, v/v), |
| --- | --- |
| sample concentration | 1.0 mg mL^–1^ |
| flow rate | 20 μL min^–1^ |
| ESI potential | 4 kV |
| bath and nebulizer gas | N_2_ |
| source temperature | 120°C |
| cone voltage range | 10–40 V |
| collision gas | argon |
| CID energy range | 5–40 eV (lab frame) |
| Typically, 10–30 scans were combined, background subtracted and smoothed to produce spectra. | |


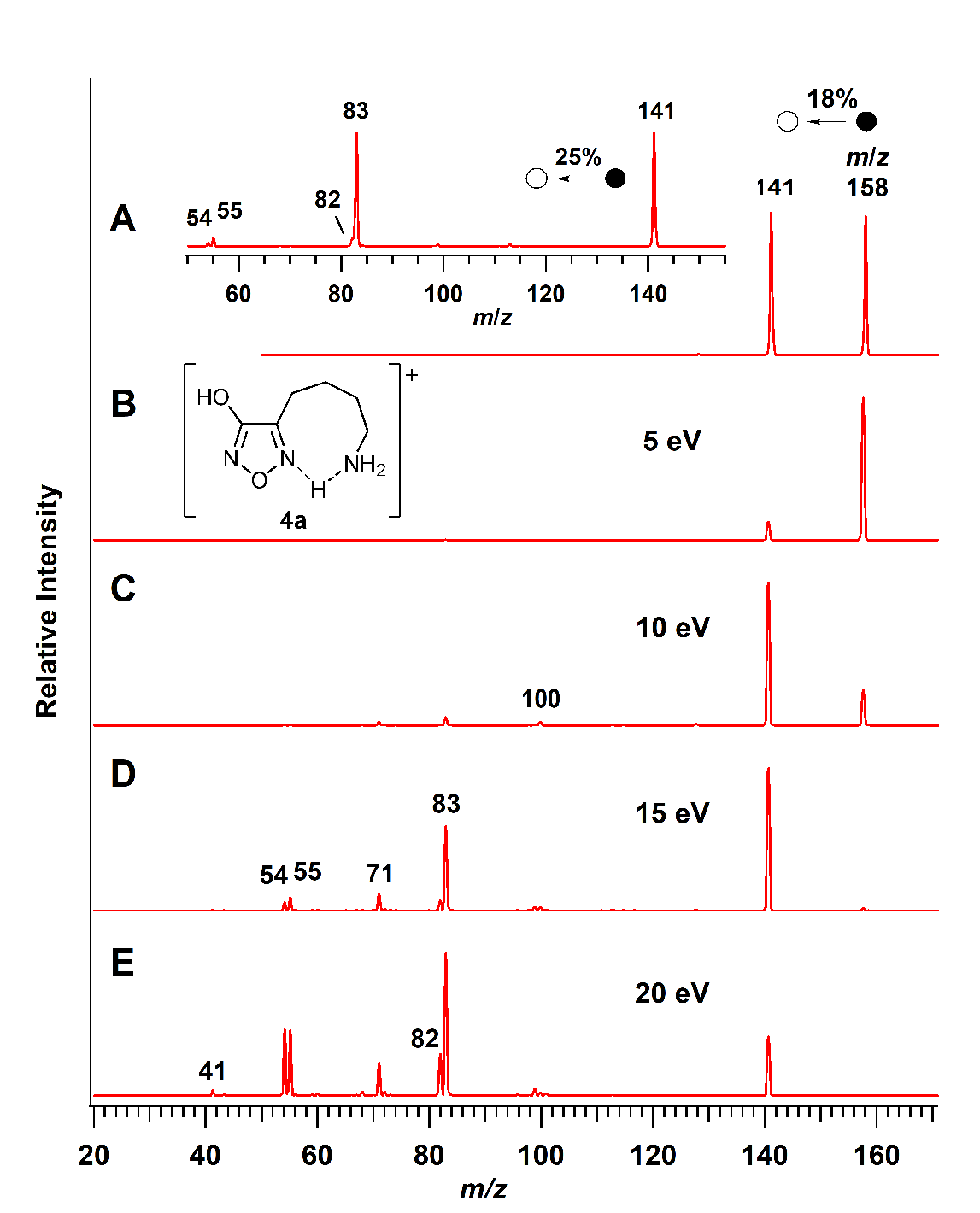
**Results**

**Figure S1.**  Tandem mass spectra of protonated 4-(4-aminobutyl)-3-hydroxyfurazan (**4a**, *m*/*z* 158, [M + H]^+^) collected on an ion trap mass spectrometer (**A**) and a triple quadrupole mass spectrometer (20 V cone) over a range of collision energies (**B–E**). For the pseudo MS^3^ spectrum (**A**, inset), the ion at *m*/*z* 141 was formed from **4a** in the source of the ion trap mass spectrometer and selected for CID.

**Table S3.** Structures computed for the 4-(ω-aminoalkyl)-3-hydroxyfurazans **3a** and **4a** varying in protonation site and conformation. Relative energies are given in kJ mol^–1^.

| Ion | **3a** |  | **4a** |
| --- | --- | --- | --- |
| Lactim  H bond to N5 | 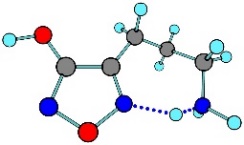**3a**(ol)  0 |  | 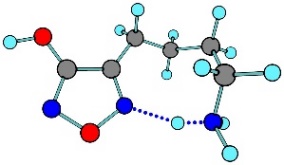**4a**(ol)  0 |
| Lactim  No H bond | 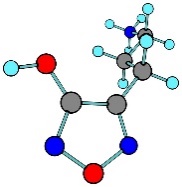**3aʹ**(ol)  50 |  | 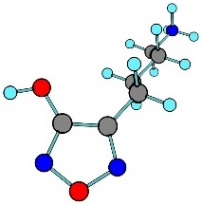  **4aʹʹ**(ol)  50 |
| Lactim  H bond to OH | Not located |  | 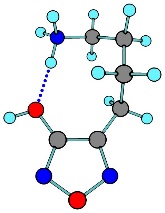  **4aʹ**(ol)  42 |
| Lactam  H bond to N5 | 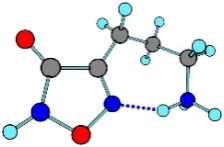**3aʹ**(one)  56 |  | **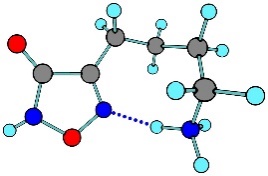4aʹ**(one)  54 |
| Lactam  No H bond | **3aʹʹ**(one)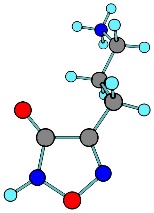  69 |  | 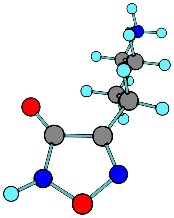**4aʹʹ**(one)  86 |
| Lactam  H bond to O | 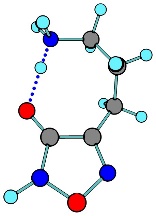**3a**(one)  15 |  | **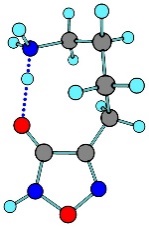4a**(one)  23 |
| N5 protomer  H bond to O | **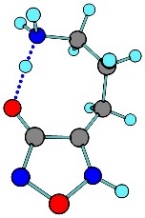3a_p_**(ol)  92 |  | **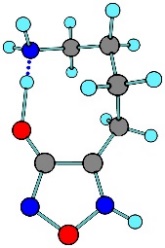4a_p_**(ol)  87 |


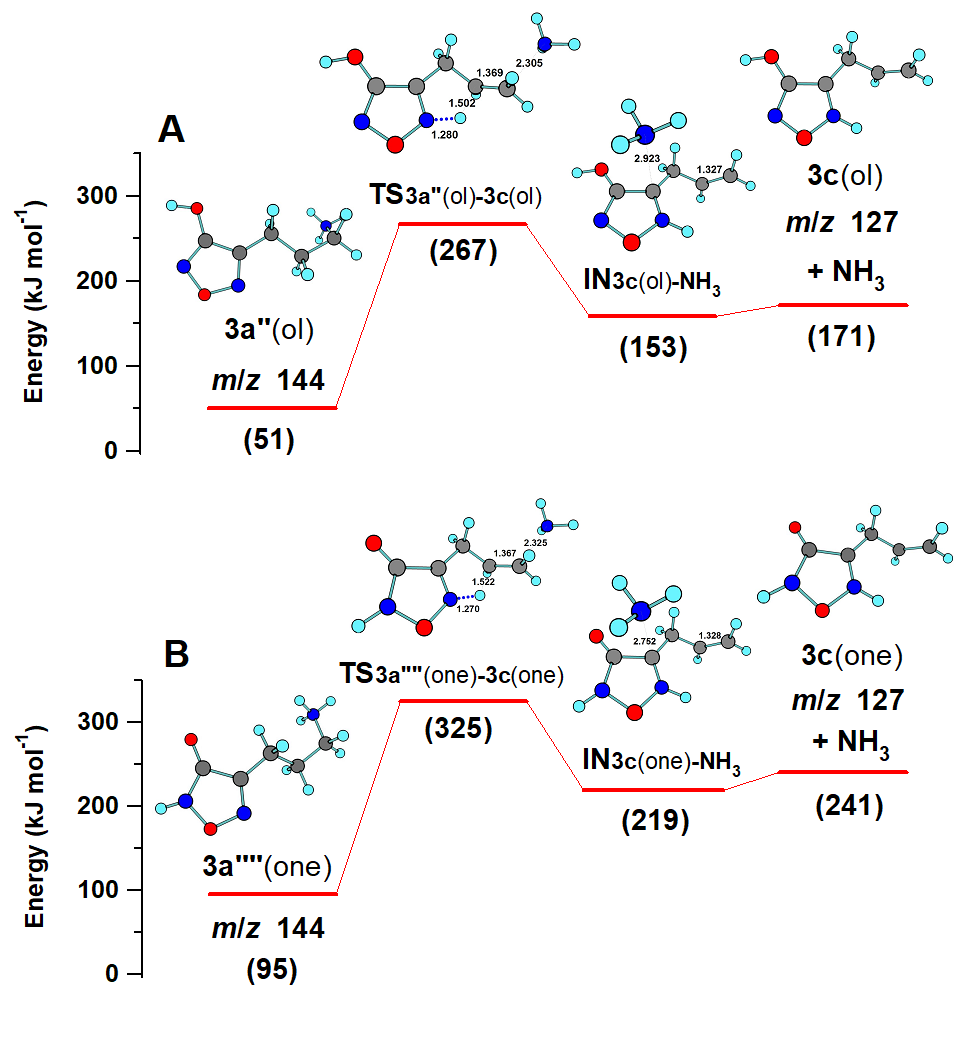


**Figure S2.** Potential energy profiles computed for the elimination of ammonia from the protonated tautomers of 4-(3-aminopropyl)-3-hydroxyfurazan **3aʹʹ**(ol) (**A**) and **3aʹʹʹʹ**(one) (**B**). Note that the side-chain proton is abstracted by N5 in the furazan ring.


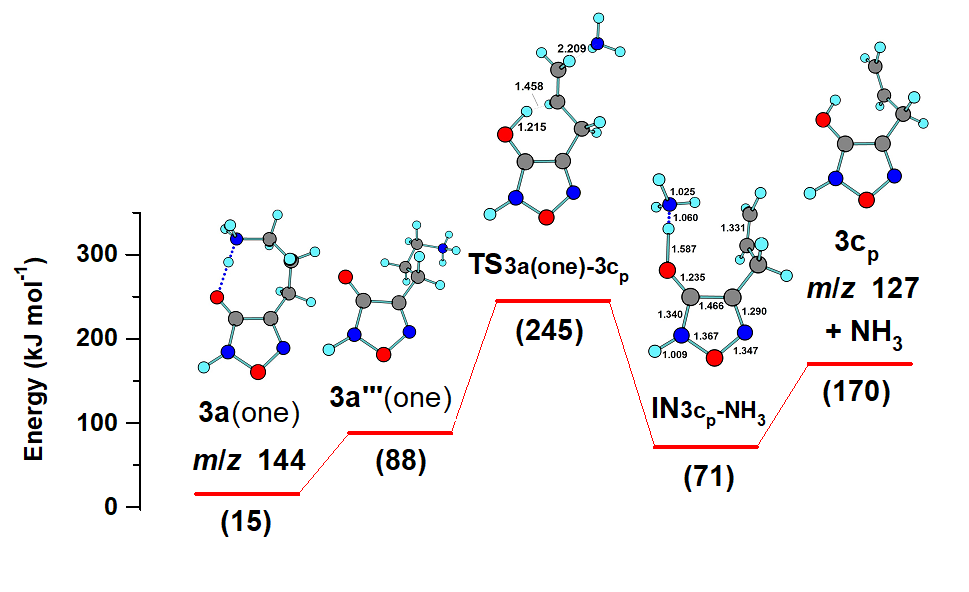


**Figure S3.** Potential energy profile computed for the elimination of ammonia from **3a**(one), the protonated lactam tautomer of 4-(3-aminopropyl)-3-hydroxyfurazan. Note that the side-chain proton is abstracted by the exocyclic, carbonyl oxygen.


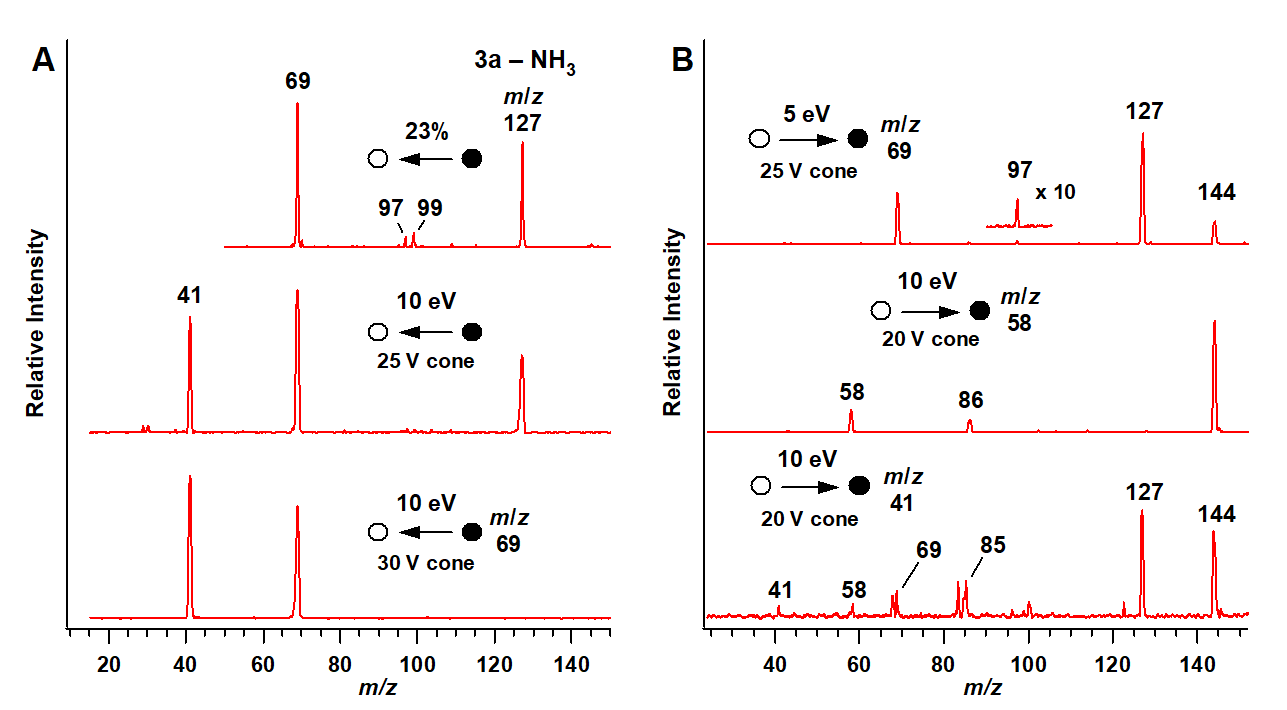


**Figure S4.**  **A:** Pseudo MS^3^ mass spectra. **B:** Precursor ion scans.

The spectra were collected for protonated 4-(3-aminopropyl)-3-hydroxyfurazan (**3a**, *m*/*z* 144, [M + H]^+^). Note that the top spectrum in **A** was collected on the ion trap mass spectrometer; all other spectra were collected on the triple quadrupole mass spectrometer.

**Scheme S1.** Parallel pathways for the fragmentation of the tautomeric ions **3a**(ol) and **3a**(one) showing an initial nucleophilic displacement of NH_3_ and sequential neutral losses of NO, CO and CH_2_=CH_2_.

**Scheme S2.** Parallel pathways for the fragmentation of the tautomeric ions **3aʹʹ**(ol) and **3aʹʹʹʹ**(one) showing sequential neutral losses of NH_3_, NO, CO and CH_2_=CH_2_. The computational results corresponding to the initial, eliminative loss of ammonia from **3aʹʹ**(ol) and **3aʹʹʹʹ**(one) are shown in Figure S2.

**Cartesian coordinates of atoms in computed structures**

**Table S3**


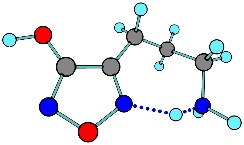


**3a**(ol) 0 kJ mol^–1^

7 -2.329461000 -1.015970000 -0.005835000

6 -1.931232000 0.217878000 0.040291000

6 -0.502313000 0.258922000 -0.009116000

7 -0.106942000 -0.975042000 -0.080390000

8 -1.201587000 -1.765796000 -0.079830000

6 0.398759000 1.448349000 0.029442000

1 -0.055299000 2.228774000 -0.587017000

1 0.393510000 1.849952000 1.049329000

8 -2.702055000 1.288911000 0.117392000

1 -3.636552000 1.050526000 0.127656000

6 1.841867000 1.231495000 -0.444180000

1 1.852992000 0.895284000 -1.487281000

1 2.324314000 2.211157000 -0.451151000

6 2.721450000 0.317961000 0.413140000

1 3.772698000 0.591440000 0.327751000

1 2.444234000 0.360389000 1.467512000

7 2.615276000 -1.125463000 -0.000470000

1 3.011359000 -1.275996000 -0.927408000

1 1.598311000 -1.385282000 -0.038717000

1 3.100273000 -1.743468000 0.648235000


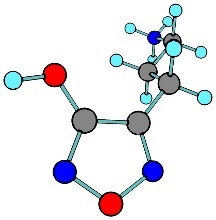


**3aʹ**(ol) 50 kJ mol^–1^

7       -2.797953000      0.433658000     -0.420478000
6       -1.635348000      0.738719000      0.061609000
6       -0.896568000     -0.453609000      0.305565000
7       -1.648257000     -1.451903000     -0.048030000
8       -2.806928000     -0.925438000     -0.486262000
6        0.485563000     -0.611607000      0.840134000
1        0.574249000     -0.052493000      1.776977000
1        0.647512000     -1.667782000      1.068269000
8       -1.184477000      1.973650000      0.287674000
1       -1.877060000      2.620633000      0.111312000
6        1.526390000     -0.108366000     -0.170314000
1        1.422719000     -0.678304000     -1.100366000
1        1.323965000      0.943473000     -0.401746000
6        2.931340000     -0.259448000      0.383342000
1        3.085991000      0.328151000      1.289212000
1        3.186403000     -1.299239000      0.592118000
7        3.948621000      0.234566000     -0.623616000
1        3.791713000      1.217868000     -0.851484000
1        3.886146000     -0.291828000     -1.496838000
1        4.904463000      0.145443000     -0.275898000


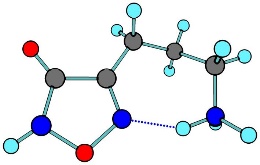


**3aʹ**(one) 56 kJ mol^–1^

7 2.349703000 -0.891920000 -0.082164000

6 1.976126000 0.427012000 -0.059523000

6 0.494636000 0.295270000 -0.001639000

7 0.163410000 -0.939239000 0.101897000

8 1.267043000 -1.730960000 0.099012000

6 -0.449400000 1.445405000 -0.034714000

1 -0.005091000 2.237377000 0.575549000

1 -0.448649000 1.848977000 -1.054414000

8 2.660190000 1.413344000 -0.094097000

6 -1.884568000 1.193332000 0.440336000

1 -1.888502000 0.864049000 1.485867000

1 -2.393546000 2.159480000 0.440928000

6 -2.736134000 0.251284000 -0.412543000

1 -3.796182000 0.487455000 -0.324712000

1 -2.463300000 0.299287000 -1.467777000

7 -2.580871000 -1.187920000 0.006256000

1 -2.973046000 -1.346938000 0.933669000

1 -1.561610000 -1.418657000 0.047708000

1 -3.046287000 -1.823784000 -0.639866000

1 3.228703000 -1.306579000 0.190303000


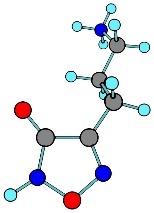


**3aʹʹ**(one) 69 kJ mol^–1^

7 -2.662397000 0.691426000 -0.262340000

6 -1.384289000 0.863878000 0.161252000

6 -0.915928000 -0.535656000 0.191585000

7 -1.853694000 -1.351626000 -0.130858000

8 -2.974505000 -0.643888000 -0.399668000

6 0.458058000 -0.951762000 0.589081000

1 0.591413000 -0.703201000 1.648080000

1 0.553545000 -2.035124000 0.488822000

8 -0.787516000 1.891165000 0.416713000

6 1.507651000 -0.205584000 -0.247438000

1 1.443654000 -0.532442000 -1.291159000

1 1.270980000 0.863469000 -0.214657000

6 2.906273000 -0.436885000 0.291592000

1 2.993518000 -0.153348000 1.341298000

1 3.245799000 -1.466926000 0.175355000

7 3.892804000 0.430400000 -0.461418000

1 3.652049000 1.418771000 -0.363880000

1 3.885295000 0.215216000 -1.459783000

1 4.848247000 0.307316000 -0.123768000

1 -3.455914000 1.312709000 -0.270782000


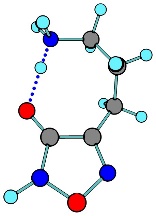


**3a**(one) 15 kJ mol^–1^

7 -2.217904000 -0.946814000 -0.133278000

6 -0.915304000 -0.810200000 0.144775000

6 -0.761892000 0.645907000 0.176120000

7 -1.893790000 1.215939000 -0.065185000

8 -2.832891000 0.269320000 -0.259124000

6 0.472240000 1.421667000 0.499040000

1 0.185925000 2.474239000 0.491745000

1 0.759595000 1.195965000 1.532179000

8 -0.084998000 -1.708394000 0.313847000

1 1.471559000 -1.412886000 0.124334000

6 1.681034000 1.219426000 -0.445642000

1 1.353247000 0.935530000 -1.451780000

1 2.166350000 2.189493000 -0.570903000

6 2.776368000 0.276800000 0.047555000

1 3.718048000 0.476970000 -0.464001000

1 2.945410000 0.394624000 1.118737000

7 2.457885000 -1.172349000 -0.182597000

1 2.516032000 -1.406782000 -1.172629000

1 3.108892000 -1.781581000 0.309973000

1 -2.819947000 -1.752015000 -0.219113000


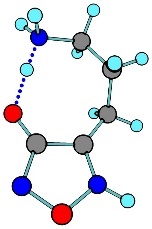


**3a_p_**(ol) 92 kJ mol^–1^

7 -2.153421000 -1.141710000 -0.140939000

6 -0.887727000 -0.851181000 0.167267000

6 -0.770901000 0.602079000 0.189495000

7 -1.961070000 1.036864000 -0.100098000

8 -2.823227000 0.024587000 -0.299907000

6 0.410263000 1.446475000 0.513585000

1 0.112867000 2.497180000 0.497480000

1 0.686063000 1.225478000 1.550683000

8 0.033387000 -1.669434000 0.406498000

1 1.412594000 -1.400696000 0.039827000

6 1.639879000 1.260491000 -0.414017000

1 1.325288000 1.016468000 -1.434226000

1 2.125809000 2.235109000 -0.490255000

6 2.724432000 0.290358000 0.058637000

1 3.678119000 0.546808000 -0.403580000

1 2.854653000 0.345007000 1.140397000

7 2.430307000 -1.135172000 -0.270863000

1 2.501346000 -1.310716000 -1.271253000

1 -2.364870000 1.961731000 -0.180877000

1 3.080468000 -1.766802000 0.192564000


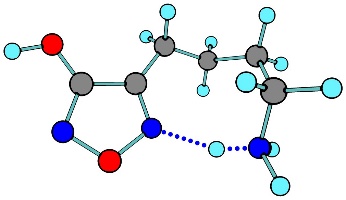


**4a**(ol) 0 kJ mol^–1^

7 2.523919000 -1.063761000 -0.050009000

6 2.169962000 0.179875000 -0.147869000

6 0.773291000 0.306224000 0.140078000

7 0.348951000 -0.890824000 0.406125000

8 1.397919000 -1.740183000 0.292462000

6 -0.060796000 1.543386000 0.171619000

1 0.496673000 2.284146000 0.751986000

1 -0.109472000 1.959185000 -0.841705000

8 2.955553000 1.195943000 -0.462119000

1 3.865779000 0.907608000 -0.597814000

6 -1.466461000 1.378446000 0.767350000

1 -1.423474000 0.704898000 1.630886000

1 -1.766099000 2.343547000 1.180843000

6 -2.583173000 0.967580000 -0.202399000

1 -3.538572000 1.001547000 0.333725000

1 -2.668853000 1.717456000 -0.994757000

6 -2.462582000 -0.377593000 -0.904203000

1 -3.344976000 -0.574738000 -1.512764000

1 -1.582566000 -0.437940000 -1.546007000

7 -2.326241000 -1.505857000 0.081552000

1 -3.007154000 -1.439519000 0.836902000

1 -2.441703000 -2.413577000 -0.366574000

1 -1.355197000 -1.463109000 0.4714120


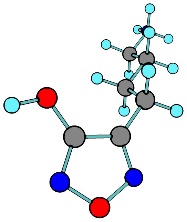


**4aʹʹ**(ol) 50 kJ mol^–1^

7 -3.347413000 0.410063000 -0.441670000

6 -2.193737000 0.737661000 0.046966000

6 -1.438030000 -0.440081000 0.314688000

7 -2.172961000 -1.453244000 -0.031740000

8 -3.336944000 -0.950162000 -0.489869000

6 -0.056127000 -0.568908000 0.857146000

1 0.026834000 0.029289000 1.770390000

1 0.107659000 -1.613685000 1.132876000

8 -1.769658000 1.983965000 0.263315000

1 -2.469934000 2.612632000 0.055526000

6 0.991457000 -0.110427000 -0.163387000

1 0.886871000 -0.708891000 -1.074310000

1 0.797242000 0.931030000 -0.440258000

6 2.412511000 -0.244732000 0.386796000

1 2.511314000 0.361307000 1.295610000

1 2.596342000 -1.288359000 0.669914000

6 3.433096000 0.198472000 -0.645156000

1 3.393407000 -0.406005000 -1.552105000

1 3.316627000 1.247809000 -0.918684000

7 4.840525000 0.054740000 -0.101067000

1 5.544336000 0.347757000 -0.780073000

1 5.038286000 -0.914842000 0.151740000

1 4.967753000 0.618715000 0.740818000


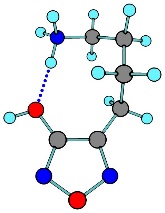


**4aʹ**(ol) 42 kJ mol^–1^

7 -2.627797000 0.985096000 0.180924000

6 -1.373831000 0.761363000 -0.023772000

6 -1.148770000 -0.625339000 -0.236170000

7 -2.307882000 -1.205547000 -0.145916000

8 -3.211608000 -0.234598000 0.103550000

6 0.143008000 -1.317959000 -0.499424000

1 -0.055367000 -2.365259000 -0.730117000

1 0.571950000 -0.886820000 -1.410882000

8 -0.429276000 1.734256000 -0.027582000

6 1.113811000 -1.194586000 0.693468000

1 0.828893000 -0.345684000 1.327829000

1 0.995890000 -2.063073000 1.343804000

6 2.588794000 -1.077726000 0.304107000

1 3.205534000 -1.084214000 1.209213000

1 2.901528000 -1.951506000 -0.275176000

1 1.404385000 1.484101000 0.085068000

6 2.938781000 0.145211000 -0.533302000

1 4.017618000 0.251671000 -0.645613000

1 2.496810000 0.122568000 -1.529220000

7 2.437603000 1.418267000 0.109254000

1 2.804980000 2.238859000 -0.371471000

1 2.729827000 1.477918000 1.085159000

1 -0.859192000 2.593687000 0.064379000


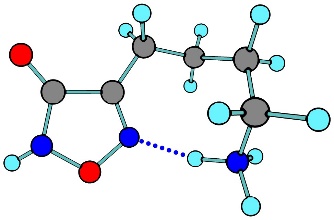


**4aʹ**(one) 54 kJ mol^–1^

7 2.525398000 -0.954859000 -0.153989000

6 2.213181000 0.382223000 -0.203658000

6 0.761870000 0.349783000 0.128145000

7 0.405709000 -0.842927000 0.436298000

8 1.460747000 -1.698792000 0.325112000

6 -0.113965000 1.551841000 0.168887000

1 0.434791000 2.304247000 0.744348000

1 -0.165929000 1.970996000 -0.843340000

8 2.920463000 1.315438000 -0.468219000

6 -1.512585000 1.352716000 0.765929000

1 -1.453269000 0.684266000 1.632957000

1 -1.837996000 2.311043000 1.175354000

6 -2.613845000 0.906955000 -0.205442000

1 -3.572615000 0.911113000 0.325586000

1 -2.718994000 1.651505000 -1.000358000

6 -2.443018000 -0.434736000 -0.902265000

1 -3.314679000 -0.667445000 -1.513667000

1 -1.558207000 -0.467421000 -1.539501000

7 -2.273994000 -1.553961000 0.091014000

1 -2.966974000 -1.509036000 0.837291000

1 -2.351748000 -2.467154000 -0.354428000

1 -1.315103000 -1.477917000 0.496350000

1 3.421428000 -1.367833000 0.061422000


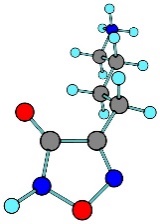


**4aʹʹ**(one) 86 kJ mol^–1^

7 -3.217877000 0.652656000 -0.336648000

6 -1.964895000 0.876924000 0.146751000

6 -1.449089000 -0.504997000 0.229995000

7 -2.349806000 -1.360273000 -0.095291000

8 -3.486140000 -0.698776000 -0.425497000

6 -0.072860000 -0.860461000 0.671360000

1 0.057969000 -0.489782000 1.694345000

1 0.028148000 -1.948062000 0.697811000

8 -1.422918000 1.930173000 0.409173000

6 0.979003000 -0.219154000 -0.241052000

1 0.900829000 -0.650007000 -1.244722000

1 0.762746000 0.849868000 -0.327545000

6 2.395289000 -0.410449000 0.302366000

1 2.449934000 0.005299000 1.315945000

1 2.628779000 -1.479759000 0.374162000

6 3.403711000 0.285931000 -0.592568000

1 3.432831000 -0.139291000 -1.596446000

1 3.211253000 1.356617000 -0.670885000

7 4.804372000 0.146440000 -0.030282000

1 5.499745000 0.606660000 -0.619131000

1 5.069927000 -0.835941000 0.054587000

1 -4.039353000 1.236160000 -0.315235000

1 4.865878000 0.558538000 0.902133000


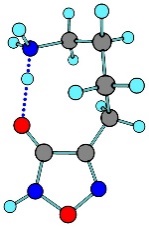


**4a**(one) 23 kJ mol^–1^

7 -2.511663000 0.996328000 0.138399000

6 -1.195944000 0.803505000 -0.011337000

6 -1.123613000 -0.647952000 -0.203575000

7 -2.301709000 -1.166183000 -0.156799000

8 -3.204737000 -0.181637000 0.051007000

6 0.135977000 -1.406417000 -0.436264000

1 -0.106367000 -2.459040000 -0.585822000

1 0.553607000 -1.055752000 -1.386474000

8 -0.301001000 1.654987000 0.015332000

6 1.149316000 -1.226261000 0.717112000

1 0.845324000 -0.396599000 1.364607000

1 1.114825000 -2.104831000 1.363680000

6 2.598654000 -1.022899000 0.266108000

1 3.247887000 -1.003891000 1.148288000

1 2.932727000 -1.878959000 -0.327910000

1 1.322763000 1.509558000 0.111500000

6 2.862507000 0.219824000 -0.575639000

1 3.929770000 0.338296000 -0.763194000

1 2.355011000 0.190453000 -1.540406000

7 2.377702000 1.469769000 0.102591000

1 2.700292000 2.303379000 -0.386106000

1 2.711624000 1.530143000 1.063568000

1 -3.073253000 1.822243000 0.279788000


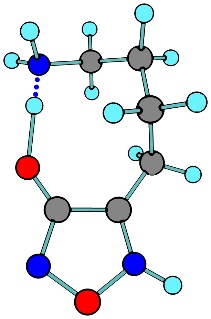


**4a_p_**(ol) 87 kJ mol^–1^

7 -2.443992000 1.194160000 0.164377000

6 -1.168459000 0.849535000 -0.016720000

6 -1.135999000 -0.598734000 -0.204212000

7 -2.373191000 -0.978259000 -0.122333000

8 -3.197214000 0.066867000 0.095363000

6 0.070629000 -1.428221000 -0.439049000

1 -0.189066000 -2.479029000 -0.579310000

1 0.488006000 -1.087321000 -1.392823000

8 -0.174414000 1.613277000 -0.032829000

6 1.099407000 -1.254323000 0.708101000

1 0.801878000 -0.422896000 1.353233000

1 1.063680000 -2.137184000 1.348874000

6 2.546757000 -1.060151000 0.244808000

1 3.196309000 -1.070475000 1.126729000

1 2.865480000 -1.911692000 -0.364140000

1 1.328321000 1.494177000 0.153393000

6 2.832913000 0.196343000 -0.570787000

1 3.900031000 0.273607000 -0.780464000

1 2.305773000 0.204359000 -1.525999000

7 2.401697000 1.439794000 0.140014000

1 2.728500000 2.274381000 -0.343409000

1 2.755778000 1.480052000 1.093910000

1 -2.834750000 -1.875701000 -0.197525000

**Figure 4A**

**3a**(ol) 0 kJ mol^–1^

see above


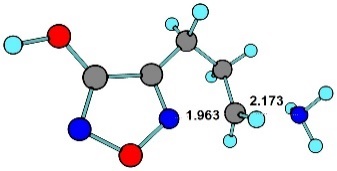


**TS3a**(ol)-**3b**(ol) 174 kJ mol^–1^

7 -2.277764000 -1.173556000 -0.007199000

6 -2.111438000 0.117266000 0.049975000

6 -0.715431000 0.407902000 -0.004216000

7 -0.124012000 -0.735067000 -0.095192000

8 -1.034594000 -1.720835000 -0.091602000

6 0.207960000 1.567825000 0.064904000

1 -0.114687000 2.387645000 -0.577603000

1 0.228144000 1.951917000 1.089135000

8 -3.067762000 1.018844000 0.142247000

1 -3.941472000 0.609426000 0.160253000

6 1.591132000 1.029323000 -0.374104000

1 1.684713000 1.104494000 -1.458764000

1 2.367196000 1.651969000 0.067113000

6 1.810315000 -0.422950000 0.028191000

1 1.828685000 -0.720295000 1.066094000

1 2.027156000 -1.181736000 -0.703341000

7 3.972992000 -0.303755000 0.200011000

1 4.287121000 0.266415000 0.979564000

1 4.403644000 0.071139000 -0.639947000

1 4.354612000 -1.234592000 0.340498000


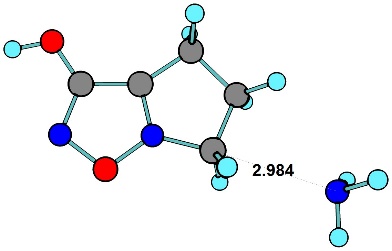


**IN3b**(ol)-NH_3_ 84 kJ mol^–1^

7 2.128470000 -1.287404000 0.009836000

6 2.148272000 0.019226000 -0.023299000

6 0.805759000 0.512439000 -0.007903000

7 0.086517000 -0.561605000 0.036441000

8 0.824905000 -1.675896000 0.041411000

6 -0.058180000 1.721054000 -0.059786000

1 0.236311000 2.459501000 0.686431000

1 0.040272000 2.193537000 -1.041001000

8 3.219074000 0.773005000 -0.062341000

1 4.033388000 0.253143000 -0.065184000

6 -1.478043000 1.119925000 0.191632000

1 -1.799772000 1.341740000 1.208251000

1 -2.220888000 1.530683000 -0.485884000

6 -1.379957000 -0.414598000 0.006541000

1 -1.748655000 -0.777775000 -0.950660000

1 -1.826683000 -0.994651000 0.808305000

7 -4.361685000 -0.349109000 -0.094284000

1 -4.778608000 0.043284000 -0.932493000

1 -4.807044000 0.124322000 0.685327000

1 -4.680372000 -1.312105000 -0.052724000


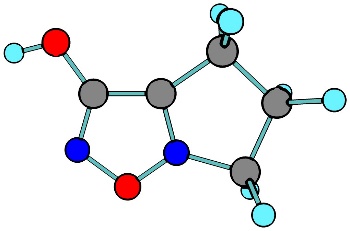


**3b**(ol) 95 kJ mol^–1^

7 -1.685160000 -1.039843000 -0.025826000

6 -1.458668000 0.248171000 -0.006969000

6 -0.046766000 0.477894000 0.013982000

7 0.455215000 -0.714999000 0.002206000

8 -0.480353000 -1.669723000 -0.012610000

6 1.031075000 1.499487000 0.084711000

1 0.902650000 2.279440000 -0.666021000

1 1.006326000 1.982910000 1.065337000

8 -2.365308000 1.191768000 -0.008911000

1 -3.265265000 0.840017000 -0.025355000

6 2.312561000 0.635684000 -0.139926000

1 2.669193000 0.765921000 -1.161245000

1 3.116030000 0.926419000 0.533577000

6 1.914149000 -0.847269000 0.083443000

**Figure 4B**

**3aʹ**(one) 56 kJ mol^–1^

see above


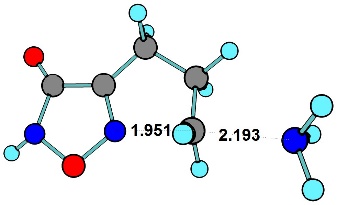


**TS3aʹ**(one)-**3b**(one) 232 kJ mol^–1^

7 2.272879000 -1.103249000 -0.092225000

6 2.191960000 0.278820000 -0.065036000

6 0.717530000 0.458644000 -0.015267000

7 0.155469000 -0.679951000 0.105914000

8 1.034118000 -1.700648000 0.107666000

6 -0.226189000 1.597511000 -0.082488000

1 0.090282000 2.420244000 0.560158000

1 -0.242489000 1.984530000 -1.106099000

8 3.081579000 1.077347000 -0.098350000

6 -1.603244000 1.036859000 0.350050000

1 -1.719044000 1.141542000 1.429931000

1 -2.387124000 1.623613000 -0.124586000

6 -1.776159000 -0.430747000 -0.014775000

1 -1.800560000 -0.752998000 -1.045497000

1 -1.979222000 -1.176121000 0.734801000

7 -3.961636000 -0.372902000 -0.180817000

1 -4.290463000 0.165050000 -0.977168000

1 -4.399048000 0.020477000 0.647224000

1 -4.326245000 -1.314744000 -0.290066000

1 3.037968000 -1.678989000 0.231765000


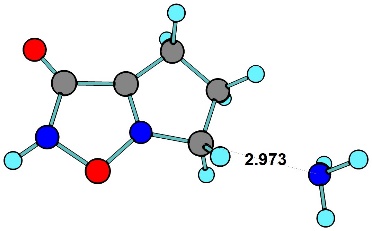


**IN3b**(one)-NH_3_ 146 kJ mol^–1^

7 2.128341000 -1.227031000 -0.100879000

6 2.244597000 0.161218000 -0.031428000

6 0.807254000 0.560396000 -0.029448000

7 0.116943000 -0.514906000 0.046116000

8 0.814276000 -1.661775000 0.054622000

6 -0.072790000 1.751957000 -0.072766000

1 0.219481000 2.485271000 0.680694000

1 0.031420000 2.238340000 -1.047162000

8 3.238941000 0.814202000 -0.007649000

6 -1.487367000 1.135903000 0.166519000

1 -1.839333000 1.382945000 1.166746000

1 -2.219073000 1.511231000 -0.542794000

6 -1.355307000 -0.399988000 0.028474000

1 -1.722105000 -0.804996000 -0.912610000

1 -1.774681000 -0.969418000 0.852840000

7 -4.326545000 -0.411592000 -0.079473000

1 -4.740726000 -0.064533000 -0.938889000

1 -4.781724000 0.096554000 0.672209000

1 -4.639548000 -1.373470000 0.007574000

1 2.791058000 -1.903561000 0.257164000


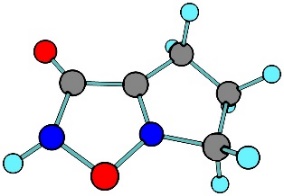


**3b**(one) 159 kJ mol^–1^

7 -1.729623000 -0.903690000 0.085055000

6 -1.512894000 0.471766000 -0.000663000

6 -0.020600000 0.519110000 0.033698000

7 0.394135000 -0.691657000 -0.012683000

8 -0.553639000 -1.641246000 -0.027792000

6 1.117967000 1.465883000 0.095727000

1 1.025658000 2.250335000 -0.656931000

1 1.119103000 1.958846000 1.072635000

8 -2.320986000 1.341710000 -0.057623000

6 2.345557000 0.528204000 -0.127688000

1 2.733560000 0.659174000 -1.137104000

1 3.150245000 0.747420000 0.570751000

6 1.843446000 -0.927562000 0.052580000

1 2.069025000 -1.371446000 1.023366000

1 2.134483000 -1.611958000 -0.742869000

1 -2.527517000 -1.413065000 -0.275056000

**Figure S2A**


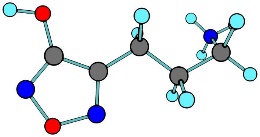


**3aʹʹ**(ol) 51 kJ mol^–1^

7 2.924430000 -0.193058000 0.148850000

6 1.941283000 0.634998000 -0.013913000

6 0.717183000 -0.092838000 -0.049489000

7 1.009082000 -1.348923000 0.095971000

8 2.347341000 -1.423994000 0.216778000

6 -0.675793000 0.416617000 -0.214820000

1 -0.874018000 1.102991000 0.620034000

1 -0.722826000 1.024353000 -1.125500000

8 2.039173000 1.955447000 -0.129417000

1 2.961610000 2.232889000 -0.090050000

6 -1.692764000 -0.724577000 -0.279577000

1 -1.428240000 -1.398220000 -1.098393000

1 -1.640646000 -1.341391000 0.623852000

6 -3.118474000 -0.273061000 -0.531223000

1 -3.208415000 0.370031000 -1.407961000

1 -3.794390000 -1.120024000 -0.647253000

7 -3.656079000 0.531819000 0.638032000

1 -3.127719000 1.394753000 0.772385000

1 -3.593797000 -0.001952000 1.506824000

1 -4.634305000 0.789248000 0.501335000


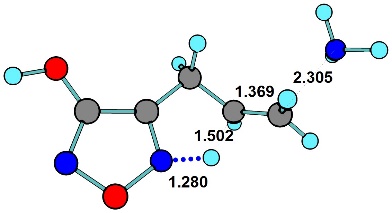


**TS3aʹ**(ol)-**3c**(ol) 267 kJ mol^–1^

7 2.784340000 -0.581658000 -0.251370000

6 2.116720000 0.527511000 -0.091568000

6 0.743400000 0.214813000 0.135397000

7 0.682790000 -1.074628000 0.097834000

8 1.893431000 -1.600955000 -0.134276000

6 -0.575921000 0.882130000 0.391139000

1 -0.490713000 1.584935000 1.221943000

1 -0.870495000 1.458546000 -0.490054000

8 2.616381000 1.744223000 -0.131947000

1 3.568538000 1.735274000 -0.290740000

6 -1.548429000 -0.255241000 0.700062000

1 -0.555561000 -1.314290000 0.316243000

1 -1.709414000 -0.478084000 1.751924000

6 -2.443696000 -0.741344000 -0.214864000

1 -2.323641000 -0.543956000 -1.273923000

1 -3.091806000 -1.566971000 0.048112000

7 -4.367897000 0.519941000 -0.359213000

1 -4.207102000 1.465022000 -0.691235000

1 -4.772818000 0.593126000 0.568241000

1 -5.072569000 0.107448000 -0.962480000


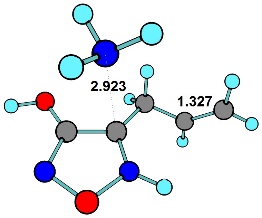


**IN3c**(ol)-NH_3_ 153 kJ mol^–1^

7 2.086262000 -0.745252000 -0.377649000

6 1.389959000 -0.201662000 0.574368000

6 -0.014221000 -0.317638000 0.295217000

7 -0.030457000 -0.958235000 -0.830742000

8 1.209632000 -1.217384000 -1.293112000

6 -1.209535000 0.137282000 1.046612000

1 -1.040657000 -0.077665000 2.106546000

1 -1.223810000 1.227998000 0.948570000

8 1.855683000 0.381387000 1.652513000

1 2.819211000 0.342653000 1.703665000

6 -2.481410000 -0.490750000 0.537406000

1 -0.801959000 -1.233028000 -1.430350000

1 -2.680461000 -1.508870000 0.864563000

6 -3.341854000 0.134701000 -0.256716000

1 -3.188528000 1.161721000 -0.577614000

1 -4.251127000 -0.350821000 -0.593045000

7 0.512841000 2.242890000 -1.011615000

1 1.274060000 2.181907000 -1.681524000

1 0.797828000 2.946014000 -0.336427000

1 -0.265238000 2.650640000 -1.520870000


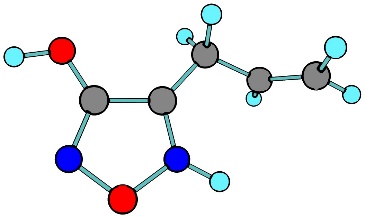


**3c**(ol) 171 kJ mol^–1^

7 2.172884000 -0.649397000 -0.052419000

6 1.471797000 0.448757000 -0.033085000

6 0.070334000 0.139546000 0.034400000

7 0.064012000 -1.160203000 0.050166000

8 1.304659000 -1.678814000 -0.000804000

6 -1.136924000 1.006057000 0.061381000

1 -0.926739000 1.842240000 0.736378000

1 -1.236267000 1.446196000 -0.938530000

8 1.927742000 1.674910000 -0.067865000

1 2.892426000 1.715998000 -0.106928000

6 -2.375680000 0.246834000 0.464496000

1 -0.703593000 -1.825799000 0.088201000

1 -2.492206000 0.049989000 1.527913000

6 -3.301724000 -0.156687000 -0.397031000

1 -3.233915000 0.052855000 -1.461463000

1 -4.184008000 -0.690091000 -0.061411000

**Figure S2B**


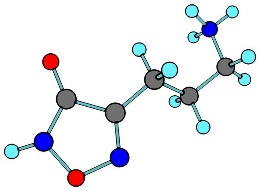


**3aʹʹʹʹ**(one) 95 kJ mol^–1^

7 2.855175000 0.118123000 0.162716000

6 1.741654000 0.878035000 -0.018849000

6 0.730539000 -0.178210000 -0.226326000

7 1.234747000 -1.350008000 -0.088835000

8 2.555593000 -1.228853000 0.172673000

6 -0.702936000 0.098335000 -0.523947000

1 -0.852652000 1.152182000 -0.255906000

1 -0.862286000 0.027039000 -1.606430000

8 1.610494000 2.080791000 -0.009264000

6 -1.657747000 -0.839948000 0.217458000

1 -1.430923000 -1.876510000 -0.043675000

1 -1.503441000 -0.767086000 1.299801000

6 -3.119053000 -0.612453000 -0.121493000

1 -3.316463000 -0.695589000 -1.191188000

1 -3.772487000 -1.303395000 0.410724000

7 -3.563196000 0.782189000 0.279570000

1 -3.056319000 1.499855000 -0.240894000

1 -3.392151000 0.950948000 1.272503000

1 -4.559141000 0.924666000 0.107834000

1 3.775343000 0.345713000 0.504741000


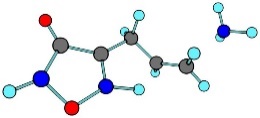


**TS3aʹʹʹʹ**(one)**-3c**(one) 325 kJ mol^–1^

7 2.746310000 -0.508318000 -0.328194000

6 2.132258000 0.713711000 -0.107446000

6 0.732087000 0.269472000 0.122588000

7 0.704923000 -1.007394000 0.132247000

8 1.897162000 -1.582270000 -0.094185000

6 -0.595989000 0.911146000 0.378428000

1 -0.506525000 1.636266000 1.190255000

1 -0.902567000 1.467672000 -0.511975000

8 2.617512000 1.805361000 -0.126027000

6 -1.546866000 -0.231585000 0.722391000

1 -0.513718000 -1.287482000 0.354890000

1 -1.704118000 -0.428057000 1.779953000

6 -2.418591000 -0.773796000 -0.180965000

1 -2.301034000 -0.604263000 -1.245115000

1 -3.055078000 -1.600308000 0.106221000

7 -4.386838000 0.446905000 -0.389744000

1 -4.241840000 1.386912000 -0.742986000

1 -4.808451000 0.535649000 0.528904000

1 -5.074316000 0.005709000 -0.992545000

1 3.722089000 -0.748869000 -0.216046000


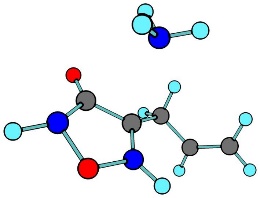


**IN3c**(one)**-NH_3_** 219 kJ mol^–1^

7 2.080133000 0.722630000 0.169313000

6 1.395918000 -0.126566000 -0.682826000

6 -0.036191000 0.154088000 -0.332997000

7 -0.014110000 1.148759000 0.475346000

8 1.220632000 1.568722000 0.858199000

6 -1.256258000 -0.445605000 -0.917956000

1 -1.081666000 -0.527644000 -1.997012000

1 -1.298867000 -1.472708000 -0.543389000

8 1.834564000 -0.886280000 -1.488421000

6 -2.503536000 0.336728000 -0.596679000

1 -0.778946000 1.613435000 0.957557000

1 -2.699901000 1.207646000 -1.218068000

6 -3.347909000 0.006628000 0.373314000

1 -3.200259000 -0.874862000 0.991809000

1 -4.239760000 0.592597000 0.565420000

7 0.559706000 -1.890897000 1.408927000

1 0.899918000 -2.673500000 0.857222000

1 -0.199833000 -2.254620000 1.976052000

1 1.307026000 -1.665828000 2.058954000

1 2.958464000 1.180874000 -0.039007000


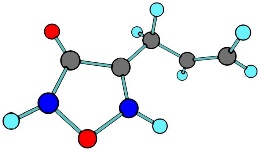


**3c**(one) 241 kJ mol^–1^

7 2.156760000 -0.548745000 -0.141609000

6 1.479390000 0.650919000 -0.047690000

6 0.052555000 0.184448000 0.028944000

7 0.098064000 -1.097237000 0.089572000

8 1.332627000 -1.643096000 0.033747000

6 -1.180201000 1.000581000 0.056639000

1 -0.985651000 1.848325000 0.724026000

1 -1.281416000 1.441724000 -0.944231000

8 1.902828000 1.762723000 -0.051413000

6 -2.396729000 0.206737000 0.458568000

1 -0.658458000 -1.777446000 0.145151000

1 -2.530533000 0.042398000 1.525384000

6 -3.284906000 -0.264274000 -0.409086000

1 -3.202001000 -0.086236000 -1.478248000

1 -4.152386000 -0.822160000 -0.074659000

1 3.122377000 -0.752207000 0.083910000

**Figure S3**

**3a**(one) 15 kJ mol^–1^

see above


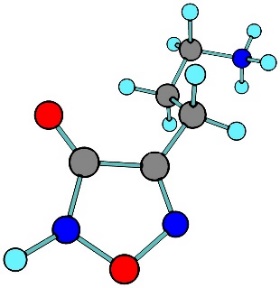


**3aʹʹʹ**(one) 88 kJ mol^–1^

7 2.781267000 0.255706000 0.223929000

6 1.580199000 0.805176000 -0.102095000

6 0.751796000 -0.411225000 -0.217425000

7 1.445602000 -1.475436000 -0.032535000

8 2.725674000 -1.122502000 0.220072000

6 -0.702322000 -0.407629000 -0.536665000

1 -0.832323000 0.061765000 -1.519383000

1 -1.026811000 -1.451062000 -0.615282000

8 1.271812000 1.969216000 -0.236625000

1 -3.340010000 -1.214521000 -0.767113000

6 -1.491134000 0.384844000 0.516496000

1 -1.440966000 -0.113330000 1.491209000

1 -1.021598000 1.364029000 0.646265000

6 -2.931914000 0.655836000 0.133288000

1 -3.451441000 1.239802000 0.892702000

1 -3.014387000 1.171944000 -0.824293000

7 -3.716596000 -0.635494000 -0.015340000

1 -3.679006000 -1.187952000 0.843022000

1 -4.699026000 -0.457662000 -0.227362000

1 3.714030000 0.637839000 0.218681000


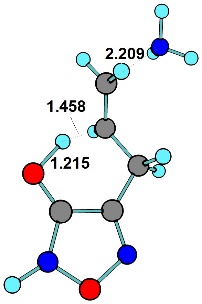


**TS3a**(one)-**3c_p_** 245 kJ mol^–1^

7 2.674861000 0.619079000 -0.058622000

6 1.360089000 0.733341000 -0.031234000

6 0.894026000 -0.640661000 -0.000020000

7 1.905441000 -1.439541000 -0.004364000

8 3.036928000 -0.703798000 -0.042346000

6 -0.564604000 -0.976456000 -0.002843000

1 -0.854166000 -1.230377000 -1.028157000

1 -0.741461000 -1.865094000 0.607755000

8 0.647689000 1.770027000 -0.020199000

1 -4.129873000 -1.407955000 -0.455608000

6 -1.368263000 0.223889000 0.496892000

1 -1.380437000 0.361873000 1.577457000

1 -0.466811000 1.315631000 0.147719000

6 -2.421229000 0.745785000 -0.218687000

1 -2.983253000 1.577627000 0.186498000

1 -2.467921000 0.625640000 -1.295155000

7 -4.256416000 -0.475514000 -0.076442000

1 -4.505320000 -0.575432000 0.902060000

1 -5.050596000 -0.059041000 -0.552455000

1 3.435589000 1.283741000 -0.078403000


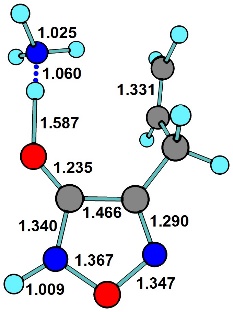


**IN3cp**-NH_3_ 71 kJ mol^–1^

7 -2.176872000 1.009443000 -0.188359000

6 -0.877014000 0.805270000 0.064117000

6 -0.825827000 -0.652538000 0.212477000

7 -2.002425000 -1.156015000 0.049341000

8 -2.879302000 -0.163186000 -0.197604000

6 0.367518000 -1.493430000 0.530172000

1 0.737587000 -1.238602000 1.528652000

1 0.024161000 -2.531729000 0.580290000

8 0.000445000 1.670836000 0.138395000

1 2.898610000 2.454438000 -0.725292000

6 1.475667000 -1.396691000 -0.487231000

1 1.171363000 -1.384467000 -1.532581000

1 1.582817000 1.784261000 0.121754000

6 2.771687000 -1.429637000 -0.184368000

1 3.528619000 -1.477711000 -0.961626000

1 3.116331000 -1.508694000 0.844931000

7 2.637988000 1.846310000 0.047585000

1 3.036053000 2.206023000 0.911831000

1 2.972869000 0.893423000 -0.126307000

1 -2.720578000 1.845852000 -0.338962000


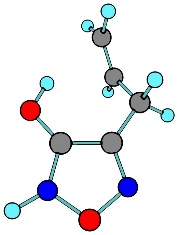


**3c_p_** 170 kJ mol^–1^

7 -1.920922000 0.803951000 -0.044436000

6 -0.613775000 0.758017000 0.044498000

6 -0.296017000 -0.648206000 0.082589000

7 -1.406986000 -1.313940000 0.006402000

8 -2.432342000 -0.453719000 -0.067720000

6 1.068742000 -1.247617000 0.217659000

1 1.305566000 -1.330685000 1.283451000

1 1.041090000 -2.262736000 -0.185539000

8 0.125188000 1.812335000 0.082327000

6 2.101284000 -0.403999000 -0.493214000

1 2.049094000 -0.390369000 -1.580473000

1 1.075734000 1.574695000 0.049935000

6 3.057402000 0.281231000 0.131221000

1 3.794019000 0.850649000 -0.425361000

1 3.170290000 0.258994000 1.212557000

1 -2.589024000 1.563892000 -0.101711000

**Figure 5**

**3b**(ol) 95 kJ mol^–1^

see above


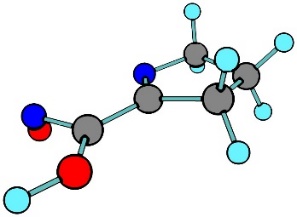


**3d_p1_ʹ** 275 kJ mol^–1^

7 2.129072000 0.518680000 -0.329564000

6 1.145973000 -0.501236000 -0.043237000

6 -0.258793000 -0.211797000 -0.073955000

7 -0.562696000 1.034028000 -0.172108000

8 2.277085000 1.431003000 0.399217000

6 -1.396785000 -1.196302000 0.007270000

1 -1.485850000 -1.737963000 -0.939800000

1 -1.260649000 -1.937611000 0.795643000

8 1.554792000 -1.704245000 0.009489000

1 2.521735000 -1.817203000 0.026878000

6 -2.570764000 -0.225723000 0.259383000

1 -3.459017000 -0.494026000 -0.309850000

1 -2.840725000 -0.224669000 1.316466000

6 -2.006717000 1.148681000 -0.147749000

1 -2.279081000 1.982959000 0.503720000

1 -2.293548000 1.463745000 -1.161269000


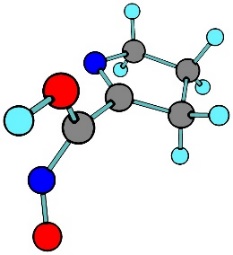


321 kJ mol^–1^

7 1.982897000 0.414199000 -0.477263000

6 1.166097000 -0.450538000 0.146820000

6 -0.284924000 -0.153656000 0.026152000

7 -0.895205000 -0.556459000 -1.002783000

8 2.529129000 1.423615000 -0.264771000

6 -1.046033000 0.645502000 1.062431000

1 -0.866769000 0.301397000 2.082740000

1 -0.765663000 1.703475000 1.013890000

8 1.548786000 -1.610292000 0.532497000

1 2.506980000 -1.741967000 0.627576000

6 -2.483626000 0.378075000 0.560716000

1 -2.953656000 -0.387226000 1.179770000

1 -3.103207000 1.272489000 0.606651000

6 -2.295530000 -0.137386000 -0.880228000

1 -2.455701000 0.630087000 -1.644634000

1 -2.935061000 -0.980995000 -1.142840000


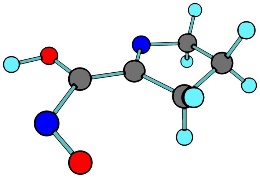


288 kJ mol^–1^

7 -2.337669000 -0.434599000 0.054190000

6 -1.161622000 0.527359000 0.041502000

6 0.230424000 0.170982000 -0.000408000

7 1.048548000 1.164313000 -0.088107000

8 -2.038886000 -1.554184000 -0.153446000

6 0.845777000 -1.210996000 0.011696000

1 0.450828000 -1.851212000 0.801088000

1 0.653890000 -1.717153000 -0.938675000

8 -1.559685000 1.719924000 0.089201000

1 -2.535438000 1.788963000 0.125854000

6 2.332899000 -0.849478000 0.204959000

1 2.630950000 -1.001130000 1.243569000

1 2.988642000 -1.453858000 -0.419295000

6 2.394759000 0.644585000 -0.152049000

1 2.735385000 0.843761000 -1.179711000

1 3.034733000 1.261997000 0.484360000


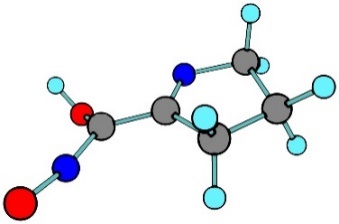


324 kJ mol^–1^

7 -1.981574000 -0.469239000 -0.052389000

6 -1.122871000 0.487661000 -0.030882000

6 0.315382000 0.133580000 0.002989000

7 1.101030000 1.113346000 0.140460000

8 -2.736179000 -1.295597000 0.191874000

6 0.897929000 -1.255055000 -0.048854000

1 0.694642000 -1.792826000 0.883998000

1 0.506342000 -1.855855000 -0.873889000

8 -1.570437000 1.705643000 -0.263677000

1 -1.928908000 2.166893000 0.507908000

6 2.392318000 -0.899199000 -0.210747000

1 3.035271000 -1.534268000 0.396488000

1 2.694690000 -1.018273000 -1.252039000

6 2.467016000 0.585173000 0.203326000

1 3.105922000 1.199360000 -0.432484000

1 2.810117000 0.732895000 1.232954000


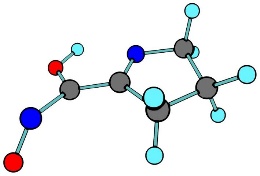


258 kJ mol^–1^

7 -2.098179000 -0.475702000 0.369574000

6 -1.122841000 0.445499000 -0.001660000

6 0.293545000 0.063945000 0.039735000

7 1.067644000 1.071378000 -0.028821000

8 -2.849453000 -1.081230000 -0.290763000

6 0.918939000 -1.297068000 0.142014000

1 0.768675000 -1.703333000 1.147520000

1 0.501652000 -2.014030000 -0.567905000

8 -1.476899000 1.660416000 -0.049418000

1 -0.668061000 2.227159000 -0.035858000

6 2.394598000 -0.932624000 -0.148927000

1 3.081939000 -1.440449000 0.525285000

1 2.658618000 -1.218073000 -1.167819000

6 2.449572000 0.602087000 0.008141000

1 3.016239000 1.119367000 -0.768200000

1 2.862627000 0.935102000 0.967336000


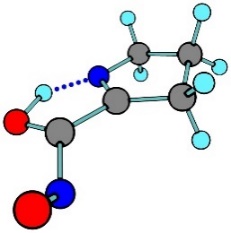


**3d_p1_** 264 kJ mol^–1^

7 -2.211348000 -0.445917000 -0.386764000

6 -1.152778000 0.396748000 0.001298000

6 0.282046000 -0.058627000 -0.035382000

7 0.992243000 0.988702000 0.026522000

8 -3.048812000 -0.883455000 0.307918000

6 1.004204000 -1.358318000 -0.122646000

1 0.641967000 -2.090188000 0.601977000

1 0.865242000 -1.792224000 -1.118198000

8 -1.273361000 1.628394000 0.029527000

1 -0.131057000 1.883971000 0.042921000

6 2.458933000 -0.883544000 0.143514000

1 2.760537000 -1.153947000 1.155710000

1 3.167217000 -1.340969000 -0.544827000

6 2.418336000 0.656227000 -0.009342000

1 2.811270000 1.018418000 -0.963526000

1 2.931503000 1.201018000 0.783430000


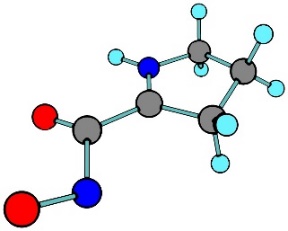


**3d** 133 kJ mol^–1^

7 2.068965000 -0.801907000 0.199138000

6 1.182332000 0.428180000 0.052331000

6 -0.293247000 0.085949000 0.062540000

7 -1.145246000 1.033444000 -0.055659000

8 3.133664000 -0.653568000 -0.284246000

6 -0.945623000 -1.242209000 0.182641000

1 -0.470231000 -1.984527000 -0.463092000

1 -0.797601000 -1.602086000 1.208888000

8 1.576433000 1.545721000 0.076786000

1 -0.861837000 2.010070000 -0.113502000

6 -2.420642000 -0.937897000 -0.163644000

1 -2.636232000 -1.245605000 -1.187054000

1 -3.112176000 -1.460902000 0.493466000

6 -2.554064000 0.589119000 -0.027752000

1 -2.984876000 0.916714000 0.921380000

1 -3.096392000 1.069493000 -0.841451000


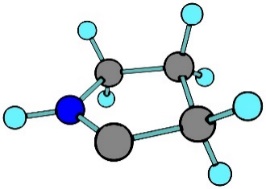


**3e** 231 kJ mol^–1^

6 -0.041180000 -1.239825000 -0.050233000

7 1.064192000 -0.625657000 -0.048574000

6 -1.257159000 -0.421407000 0.078784000

1 -2.015315000 -0.691726000 -0.657247000

1 -1.688178000 -0.596057000 1.070762000

1 1.973734000 -1.075353000 -0.095365000

6 -0.651430000 1.004807000 -0.108556000

1 -0.862996000 1.358116000 -1.117309000

1 -1.082920000 1.711112000 0.597877000

6 0.872184000 0.853078000 0.096764000

1 1.216728000 1.131093000 1.093743000

1 1.475120000 1.362499000 -0.652993000

**Neutral Molecules**

**NO**

7 0.000000000 0.000000000 -0.609416000

8 0.000000000 0.000000000 0.533239000

**CO**

6 0.000000000 0.000000000 -0.643193000

8 0.000000000 0.000000000 0.482394000


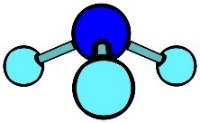


**NH_3_**

7 0.000000000 0.000000000 0.107895000

1 0.000000000 0.945970000 -0.251755000

1 -0.819234000 -0.472985000 -0.251755000

1 0.819234000 -0.472985000 -0.251755000
